# Therapeutic Peptide‐Programmed Liquid‐Crystalline Topology Switching for pH‐Gated Postoperative Cancer Immunotherapy

**DOI:** 10.1002/advs.78360

**Published:** 2026-10-18

**Authors:** Yan Yan, Bin Chen, Yanqing Wang, Yuxi Hou, Zhenlin Ma, Zini Yang, Xia Yin, Yiling Chen, Litao Huang, Menghan Cai, Xinlin Liu, Yi Zhun Zhu, Li Ye

**Affiliations:** ^1^ School of Pharmacy and Laboratory of Drug Discovery from Natural Resources and Industrialization Macau University of Science and Technology Macau China; ^2^ Livzon Pharmaceutical Group Inc. National Engineering Research Center of Microsphere Technology for Controlled‐Release Drug Delivery Zhuhai China; ^3^ Cancer Institute of the Affiliated Hospital of Qingdao University Qingdao University Qingdao China

**Keywords:** immunogenic cell death, liquid‐crystalline depots, pH‐gated release, postoperative cancer immunotherapy, therapeutic peptides, topological phase transitions

## Abstract

Stimuli‐responsive depots for postoperative cancer therapy often treat therapeutic payloads as passive cargo, resulting in premature leakage and limited on‐demand activation. Herein, we report a therapeutic peptide‐programmed liquid‐crystalline depot in which the Vpr‐derived antitumor peptide (VDAP) functions simultaneously as an immunotherapeutic effector and a supramolecular topology regulator. At physiological pH, cationic α‐helical VDAP was electrostatically anchored by oleic acid‐containing lipid domains, reinforcing a compact inverse hexagonal (*H*_II_) topology associated with reduced peptide diffusion. In the acidic postoperative tumor microenvironment, protonation of oleic acid could disrupt anionic anchoring, whereas histidine protonation within VDAP amplified intrachannel electrostatic repulsion. This cooperative peptide–lipid charge reprogramming could drive graded liquid‐crystalline topology remodeling from a diffusion‐restrictive *H*_II_ organization to a more interconnected bicontinuous cubic *Im*3̄*m* network, consistent with increased pH‐responsive peptide release. In an orthotopic incomplete‐resection breast tumor model, the depot induced immunogenic tumor destruction, enhanced damage‐associated molecular pattern release, promoted dendritic cell maturation and CD8^+^ T cell infiltration, and reshaped the immunosuppressive surgical bed. These results demonstrate that electrostatic‐topology‐gated VDAP‐enabled supramolecular topology switching provides effective adaptive local cancer immunotherapy, effectively suppressing postoperative tumor recurrence and attenuating pulmonary metastasis.

## Graphical abstract

A VDAP‐programmed liquid‐crystalline depot enables pH‐responsive postoperative immunotherapy for triple‐negative breast cancer. VDAP stabilizes a leakage‐resistant *H*_II_ mesophase through peptide–lipid electrostatic assembly, whereas tumor acidosis unlocks the matrix and promotes graded topology remodeling toward a bicontinuous *Im*3̄*m* cubic phase for accelerated release. The depot converts local tumor destruction into systemic antitumor immunity, suppressing recurrence and pulmonary metastasis.

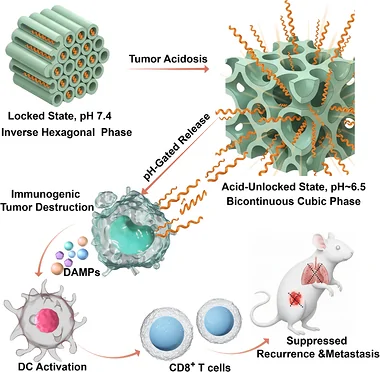

## 1 Introduction

Postoperative recurrence and metastasis remain major barriers to achieving durable treatment responses in triple‐negative breast cancer (TNBC), driven largely by persistent residual tumor cells post‐resection [1, 2, 3]. It is now understood that the postsurgical niche is not merely a passive wound environment; it is frequently characterized by inflammation, hypoxia, acidosis, and immunosuppression, which collectively promote residual tumor regrowth and distant dissemination [4, 5]. Local immunotherapy offers an attractive strategy to concentrate therapeutic agents at the resection site while reducing systemic toxicity. However, an effective postoperative depot must fulfill two competing requirements: early‐phase payload retention to prevent leakage and rapid, microenvironment‐triggered release [6, 7, 8].

Over the past few years, in situ‐forming soft depots, including hydrogels, lipid gels, and other stimuli‐responsive matrices, have been widely explored for localized cancer therapy [9, 10, 11, 12]. Although these systems can improve local retention, their release kinetics often depend on passive diffusion, matrix swelling, degradation, or bio‐orthogonal responsive motifs [13, 14, 15]. As a result, many depots suffer from either premature burst release under physiological conditions or delayed activation upon pathological stimulation [8, 14]. A major limitation is that therapeutic payloads are commonly treated as passive cargos physically entrapped within a predesigned carrier [12, 16]. Such material–payload decoupling overlooks the possibility to harness the intrinsic charge, amphiphilicity, or secondary structure of a therapeutic molecule to actively regulate the supramolecular organization of the depot itself [16, 17, 18].

Lyotropic liquid crystals (LLCs) provide a particularly suitable platform to address this limitation given that their ordered nanostructures are highly sensitive to molecular packing and interfacial interactions [19, 20, 21, 22]. Amphiphilic lipids can self‐assemble into nonlamellar mesophases, including inverse hexagonal (*H*_II_) and bicontinuous cubic (*Im*3̄*m*) phases, whose distinct topologies strongly influence molecular transport [23, 24]. In principle, switching between these mesophases could convert a compact diffusion‐restrictive structure into a more open interconnected network for controlled release. In practice, however, conventional LLC depots function as relatively static diffusion barriers since the critical packing parameter of the lipid network is not dynamically reprogrammed in situ [25]. While additives can introduce environmental responsiveness, they may also destabilize mesophase organization or act independently of the therapeutic payload [26, 27]. Therefore, designing therapeutic payloads that actively participate in LLC topological regulation represents an underexplored strategy for adaptive local delivery.

Therapeutic peptides are well positioned for such a design, yet they are typically considered challenging payloads given their susceptibility to enzymatic degradation, rapid diffusion, and strong interactions with biological membranes [28, 29, 30]. Herein, we focused on a Vpr‐derived antitumor peptide, VDAP, a cationic amphipathic α‐helical peptide previously identified as an inducer of immunogenic tumor cell death [31]. Rather than treating its cationic, membrane‐active features solely as pharmaceutical liabilities, we sought to repurpose these physicochemical properties as supramolecular design elements. We hypothesized that VDAP by virtue of its cationic, amphipathic, and histidine‐containing pH‐responsive features, could serve not only as an immunotherapeutic effector but also as a peptide regulator capable of programming the electrostatic and topological state of a lipidic liquid‐crystalline depot.

To implement this concept, oleic acid (OA) was incorporated into a sorbitan monooleate/soybean phosphatidylcholine (SMO/SPC)‐based LLC matrix as a protonatable electrostatic switch [32, 33, 34]. It is well‐ established that at physiological pH, deprotonated OA provides anionic anchoring sites for cationic VDAP, promoting peptide–lipid co‐assembly and stabilizing a compact *H*_II_ ‐type liquid‐crystalline topology [35, 36, 37]. This electrostatically locked state minimizes premature peptide diffusion [38, 39]. In contrast, in the acidic postoperative tumor microenvironment, protonation of OA reportedly weakens the anionic anchoring [40, 41], while protonation of histidine residues in VDAP amplifies repulsive interactions within confined aqueous channels [42, 43]. This cooperative VDAP–lipid charge reprogramming was designed to drive graded topological transition from the diffusion‐restrictive *H*_II_ phase toward a more interconnected bicontinuous cubic *Im*3̄*m* network, thereby converting microenvironmental acidity into electrostatic‐topology‐gated peptide release.

Herein, we report a therapeutic peptide‐programmed liquid‐crystalline depot for pH‐gated postoperative cancer immunotherapy (Figure 1). In this system, VDAP is designed to act beyond a passive payload as a supramolecular regulator that links peptide–lipid electrostatics, liquid‐crystalline topology remodeling, and on‐demand therapeutic release. Our findings substantiated that this electrostatic‐topology‐gated depot could enable localized VDAP activation in the acidic postoperative tumor microenvironment, promote immunogenic tumor destruction and antitumor immune activation, and suppress both local recurrence and pulmonary metastasis in an incomplete TNBC resection model. Overall, this work positions therapeutic peptides as active supramolecular regulators for constructing adaptive liquid‐crystalline depots that couple microenvironmental cues with controlled local immunotherapy after tumor surgery.

**FIGURE 1:**
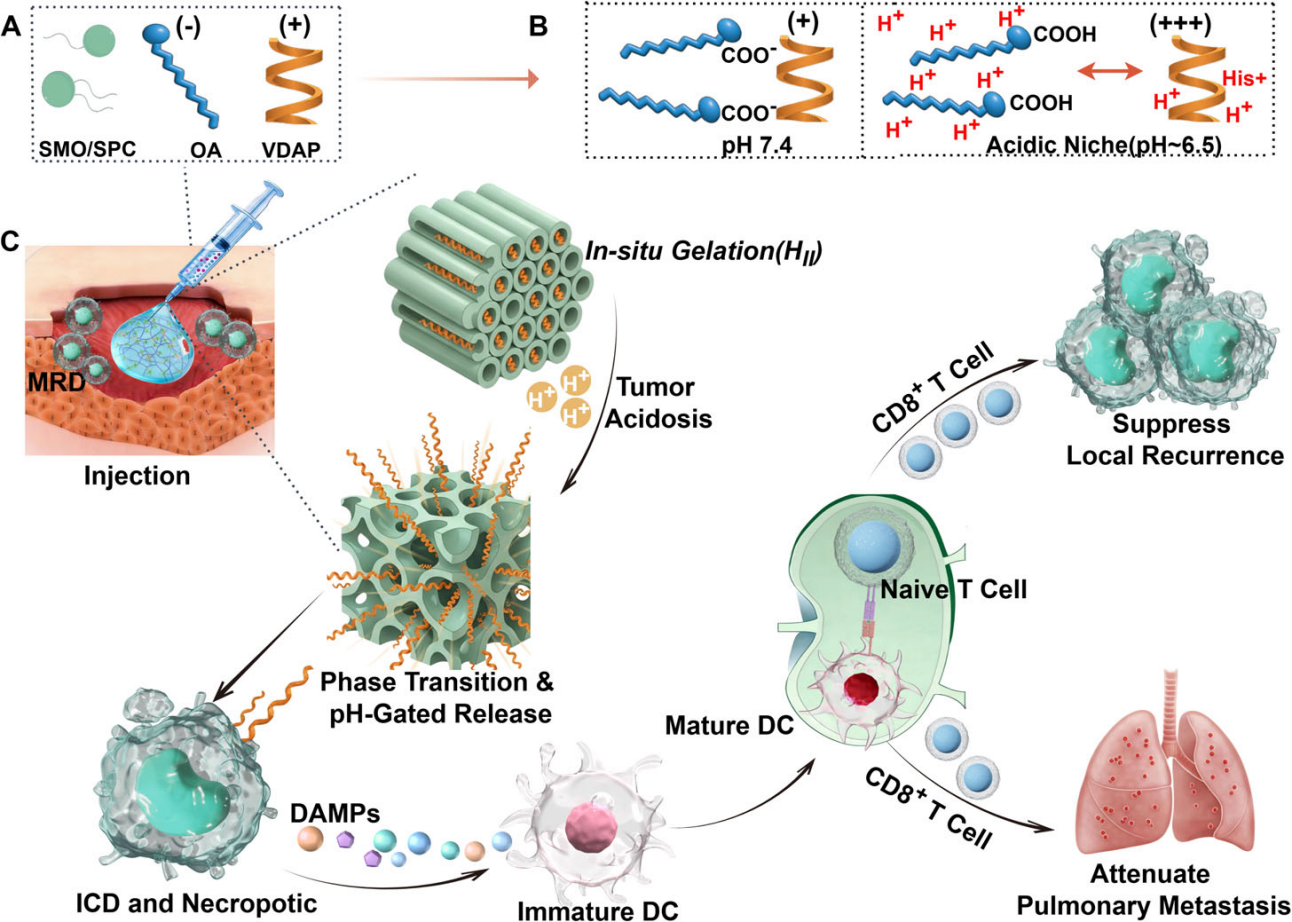
Schematic illustration of therapeutic peptide‐programmed liquid‐crystalline topology switching for pH‐gated postoperative cancer immunotherapy. (A) Molecular components of the peptide‐programmed depot, including the SMO/SPC‐based lyotropic liquid‐crystalline matrix, protonatable oleic acid (OA), and the cationic therapeutic peptide VDAP. (B) pH‐dependent electrostatic regulation between OA and VDAP. At physiological pH 7.4, deprotonated OA electrostatically anchors cationic VDAP, establishing a peptide–lipid locked state. In the acidic postoperative tumor microenvironment, OA protonation and VDAP histidine protonation cooperatively weaken peptide–lipid anchoring and increase intrachannel electrostatic repulsion. (C) After local injection into the postsurgical tumor bed, the precursor depot undergoes in situ gelation into a compact *H*_II_‐type liquid‐crystalline structure. Acid‐triggered charge reprogramming promotes a topology transition toward a more interconnected bicontinuous cubic *Im*3̄*m* network, enabling on‐demand VDAP release. The released peptide induces immunogenic and necroptotic tumor cell death, enhances DAMP release, promotes dendritic cell maturation and CD8^+^ T cell infiltration, and ultimately suppresses postoperative tumor recurrence and pulmonary metastasis. Abbreviations: SMO, sorbitan monooleate; SPC, soybean phosphatidylcholine; OA, oleic acid; LLC, lyotropic liquid crystal; ICD, immunogenic cell death; DAMPs, damage‐associated molecular patterns.

## 2 Results and Discussion

### 2.1 Programming Liquid‐Crystalline Topology Through Peptide–Lipid Electrostatic Coupling

To reconcile stable local retention with on‐demand peptide activation, we engineered a VDAP‐programmed lyotropic liquid‐crystalline depot that exploits this cationic amphipathic peptide as both a therapeutic payload and a supramolecular regulator. In this design, electrostatic coupling between cationic VDAP and protonatable OA within the SMO/SPC matrix was expected to lock the depot into a diffusion‐restrictive *H*_II_‐type topology at physiological pH, whereas acid‐induced charge reprogramming would relax this topology to enable pH‐gated release.

#### 2.1.1 Establishing an Injectable and Mechanically Adaptive LLC Depot for Peptide‐Programmed Topology Switching

To achieve pH‐gated topology switching in a postsurgical tumor bed, the liquid‐crystalline depot was designed to balance structural stability against dynamic responsiveness, resisting premature aqueous disintegration and maintaining injectability while retaining capacity for peptide‐ and pH‐coupled reorganization [44, 45]. Formulation boundary conditions were thus systematically defined to establish stable depot formation.

OA was introduced into the SMO/SPC‐based lyotropic liquid‐crystalline matrix as a protonatable lipid component that modulates both interfacial curvature and electrostatic coupling with VDAP. A compositional gradient with increasing OA content was screened to identify the structural window required for depot integrity (F0–F3; Table S1). Upon hydration, formulations lacking or containing insufficient OA rapidly lost macroscopic integrity, whereas the OA‐enriched matrix maintained a coherent depot structure (Figure 2A). This observation indicates that OA is not merely a formulation additive but a key structural component that reinforces inverse‐curvature lipid assembly in the solvent‐containing precursor system [46, 47]. By increasing the critical packing parameter of the lipid mixture, OA could promote the formation of non‐lamellar mesophases required for a mechanically retained local depot.

**FIGURE 2:**
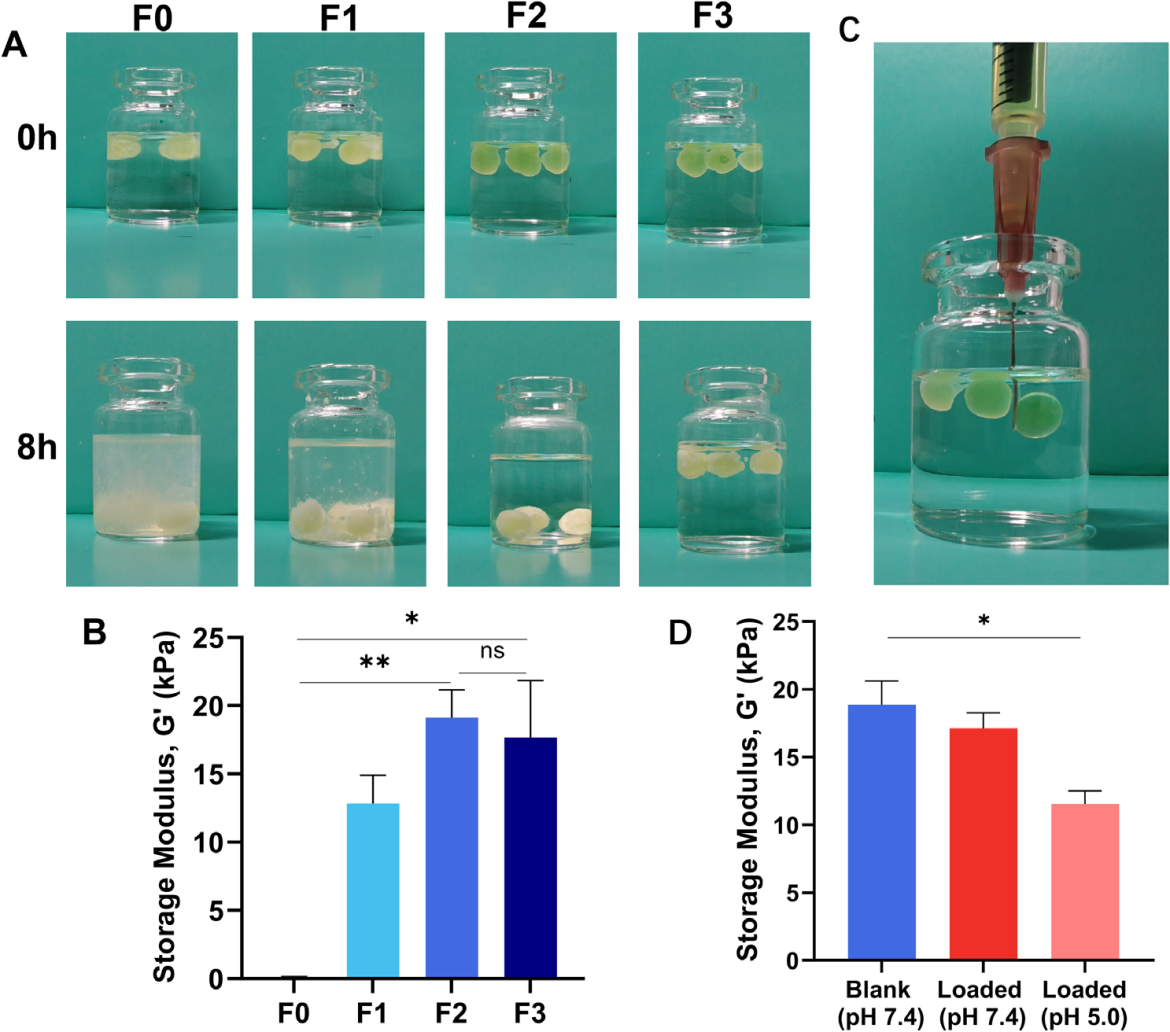
Screening and rheological characterization of structurally dynamic LLC precursors. (A) Macroscopic stability of lipid precursors with increasing OA ratios (F0–F3) after injection into excess PBS (pH 7.4) and incubation at 37°C for 8 h. (B) Plateau storage modulus (G') of formulations F0–F3, highlighting the OA‐dependent stiffening effect. (C) Macroscopic injectability and in situ gelation of the optimized F2 precursor loaded with VDAP after extrusion through a 26G needle into PBS (pH 7.4). (D) Plateau G' of the blank F2 matrix at pH 7.4, VDAP‐loaded matrix at pH 7.4, and VDAP‐loaded matrix at pH 5.0, demonstrating payload‐ and pH‐responsive rheological modulation. Data are presented as mean ± SD (*n* = 3). Statistical significance was determined using the Kruskal–Wallis test followed by Dunn's multiple comparisons test. In (B), *p < 0.05 and **p < 0.01 vs. F0; no significant difference was observed between F2 and F3. In (D), *p < 0.05 vs. the blank F2 matrix at pH 7.4.

Rheological analysis further confirmed the mechanical reinforcement conferred by OA. The optimized OA‐containing formulation, F2, displayed a solid‐like viscoelastic profile with a plateau storage modulus *G'* of 19.1 kPa, representing an approximately 190‐fold increase compared to the highly fluidized F0 matrix (Figure 2B and Figure S1A). This pronounced increase in *G'* suggests that OA establishes the mechanical foundation for depot retention rather than producing a nonspecific viscosity enhancement. Further increasing the OA content yielded no additional structural advantage, thereby defining F2 as the formulation window that balances matrix cohesion and processability.

The precursor formulation also exhibited the flow behavior required for minimally invasive local administration. In vitro injectability testing showed smooth extrusion through a 26G needle, while steady‐shear rheology confirmed pronounced shear‐thinning behavior (Figure 2C and Figure S2). Upon exposure to an aqueous environment, the injected precursor underwent spontaneous in situ gelation to form a coherent semi‐solid depot. These properties establish the suitability of the formulation as an injectable postsurgical depot capable of sustained local retention after administration.

Subsequent rheological characterization assessed whether incorporation of VDAP altered the macroscopic mechanics of the optimized LLC matrix. As expected, loading VDAP moderately decreased the plateau *G'* from 19.1 to 15.5 kPa (Figure 2D and Figure S1B). Rather than indicating matrix destabilization, this moderate softening is consistent with peptide–matrix coupling, in which the amphipathic cationic VDAP interacts with the lipid–water interface instead of behaving as a passive cargo confined within aqueous domains [48, 49]. Such peptide‐induced mechanical modulation provides macroscopic confirmation that VDAP directly participates in the supramolecular organization of the depot.

Environmental acidification further revealed the dynamic nature of the VDAP‐integrated matrix. Exposure to pH 5.0 (employed as a strongly acidic boundary condition to probe maximal structural relaxation) induced an additional decrease in *G'* while maintaining a solid‐like modulus above 10 kPa (Figure 2D). This mechanical response suggests that acidification promotes internal structural reorganization without causing macroscopic depot collapse. Mechanistically, protonation of OA weakens the anionic anchoring sites for VDAP, while protonation of histidine residues in VDAP increases cationic charge density and intrachannel electrostatic repulsion [50]. The resulting mechanical softening therefore provides macroscopic evidence for pH‐coupled charge reprogramming within the depot.

Collectively, these results establish F2 as an injectable and mechanically adaptive LLC matrix that combines aqueous stability, local retention, peptide–matrix coupling, and acid‐responsive structural relaxation. The optimized VDAP‐integrated formulation is hereafter denoted as VDAP‐LLC, and the corresponding peptide‐free matrix as Blank LLC. These macroscopic findings collectively provide the formulation and mechanical basis for the electrostatic gating, liquid‐crystalline topology switching, and pH‐triggered release behaviors investigated in the following sections.

#### 2.1.2 pH‐Dependent Electrostatic Reprogramming of the VDAP‐OA Interface

To determine whether VDAP was electrostatically coupled to the OA‐containing LLC matrix, the pH‐dependent surface charge evolution of the depot was first examined. Our findings revealed that VDAP possessed an amphipathic α‐helical structure, in which hydrophobic residues favored interfacial insertion, whereas cationic residues provided electrostatic affinity toward anionic lipid components (Figure 3A) [51, 52]. At physiological pH 7.4, deprotonated carboxylate groups of OA served as anionic anchoring sites for cationic VDAP, thereby promoting peptide–lipid association within the LLC interface.

**FIGURE 3:**
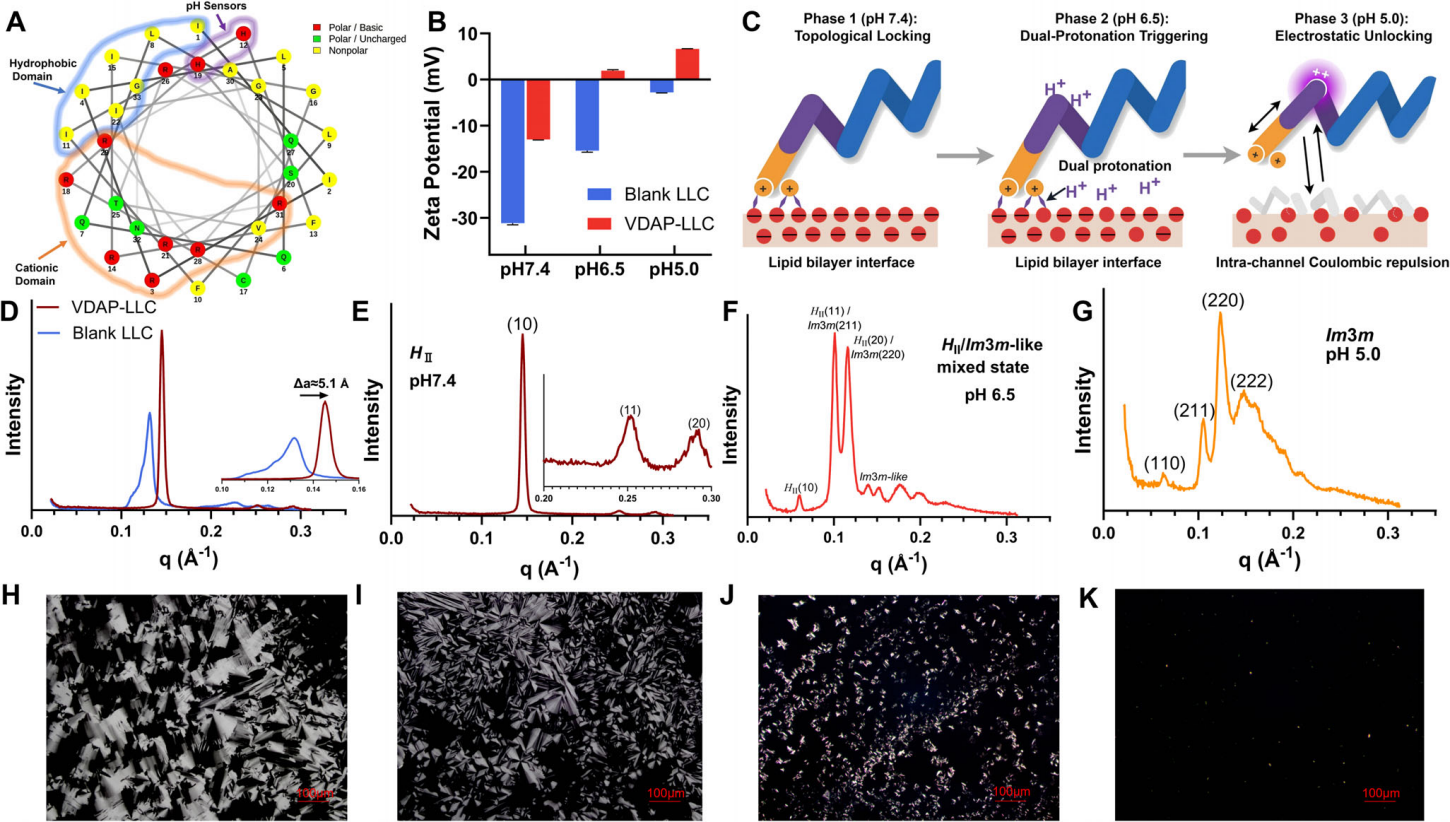
pH‐triggered supramolecular topology switching and interfacial characterization of VDAP‐LLC. (A) Helical wheel projection of VDAP showing its amphipathic organization, including the hydrophobic domain (blue), cationic anchoring domain (orange), and histidine‐containing pH‐sensing residues (purple). (B) Zeta potential profiles of Blank LLC and VDAP‐LLC under different pH conditions. Data are presented as mean ± SD, *n* = 3. Pairwise comparisons between Blank LLC and VDAP‐LLC at each pH value were performed using Mann–Whitney tests with correction for multiple comparisons; no significant differences were observed. (C) Schematic illustration of the pH‐dependent cooperative electrostatic switching mechanism. (D–G) Small‐angle X‐ray scattering profiles. (D) Superimposed 1D scattering profiles at pH 7.4. The inset shows contraction of the lattice parameter from 55.1 to 50.0 Å after VDAP loading. (E) VDAP‐LLC at pH 7.4 showing characteristic 1: √3: 2 reflections of the inverse hexagonal *H*_II_ mesophase. (F) VDAP‐LLC at pH 6.5 showing an intermediate scattering pattern. The first three peak‐position ratios (1, 1.690, and 1.940) are close to the characteristic 1: √3: 2 reflections of the *H*_II_ phase, whereas additional higher‐q features (2.333, 2.940, and 3.833) approximate *Im*3̄*m*‐related reflections, indicating a mixed *H*_II_/cubic‐like intermediate state under weakly acidic conditions. (G) VDAP‐LLC at pH 5.0 showing √2: √6: √8: √12 reflections of the primitive bicontinuous cubic *Im*3̄*m* phase. The theoretical √4 (200) reflection is suppressed by the bilayer form‐factor minimum. (H–K) Polarized light microscopy (PLM) images of (H) Blank LLC at pH 7.4, (I) VDAP‐LLC at pH 7.4 showing anisotropic focal‐conic textures, and (J) VDAP‐LLC at pH 6.5 showing attenuated but still detectable birefringent textures, and (K) VDAP‐LLC at pH 5.0 showing an optically isotropic dark field. Scale bar: 100 µm.

Zeta potential measurements supported this electrostatic coupling. Blank LLC exhibited a strongly negative surface potential of −31.1 mV, whereas the incorporation of VDAP attenuated the negative charge to −13.0 mV (Figure 3B). This charge compensation indicates electrostatic interactions between cationic VDAP and deprotonated OA, establishing an interfacial anchoring state that restricts premature peptide diffusion under physiological conditions [53, 54].

The VDAP/OA interface further displayed pronounced pH responsiveness. When the pH was decreased to 6.5, a mildly acidic condition representative of the tumor microenvironment, the zeta potential of VDAP‐LLC shifted to +1.9 mV, indicating substantial attenuation of the initial anionic character and partial electrostatic unlocking. At pH 5.0 (used to probe the maximal electrostatic response of the system), the zeta potential further shifted to +6.6 mV (Figure 3B). This pH‐dependent charge reversal is consistent with cooperative protonation at the peptide–lipid interface: protonation of OA reduces the density of anionic anchoring sites, while protonation of histidine residues in VDAP increases the local cationic charge density. According to the Henderson–Hasselbalch relationship, histidine residues with pKa values around 6.0 undergo a marked increase in protonation as the pH decreases from neutral to acidic conditions, thereby enhancing intrachannel electrostatic repulsion. Taken together, these results demonstrate pH‐dependent electrostatic reprogramming of the VDAP‐OA interface, as schematically illustrated in Figure 3C.

#### 2.1.3 Electrostatic Reprogramming Drives Graded H_II_‐to‐ Im3̄ m Topology Switching

To determine whether pH‐dependent electrostatic reprogramming at the VDAP‐OA interface induces structural reorganization of the LLC matrix, the liquid‐crystalline phase behavior was characterized across physiological and acidic conditions using Small‐Angle X‐ray Scattering (SAXS) and Polarized Light Microscopy (PLM). Here, pH 7.4 represented the physiological locked state, pH 6.5 approximated the weakly acidic postoperative tumor microenvironment, and pH 5.0 served as a strongly acidic condition to define the maximal structural response of the VDAP‐programmed LLC matrix. At pH 7.4, SAXS profiles showed that the matrix retained a well‐defined inverse hexagonal *H*_II_ phase (Figure 3D,E). Notably, VDAP loading induced a contraction of the *H*_II_ lattice, with the lattice parameter decreasing from 55.1 Å to 50.0 Å. This lattice contraction indicates that VDAP does not merely occupy the aqueous domains as a passive payload. Instead, electrostatic pairing between cationic VDAP and deprotonated OA appears to reduce interfacial charge repulsion and promote a more compact inverse‐curvature assembly. This condensed *H*_II_ topology restricted VDAP within discrete, non‐interconnected one‐dimensional aqueous channels, contributing to a diffusion‐restrictive locked state.

At pH 6.5, SAXS profile retained *H*_II_ ‐related reflections, indicating residual hexagonal ordering, alongside broadened scattering features consistent with emerging *Im*3̄*m* ‐like cubic symmetry at higher *q* values (Figure 3F). These features suggest partial structural rearrangement into a coexistent intermediate state combining *H*_II_‐like ordering with cubic‐like characteristics under mildly acidic conditions. Upon further acidification to pH 5.0, the structural transition became more pronounced. At pH 5.0, SAXS analysis revealed complete conversion from the compact *H*_II_ phase to a bicontinuous cubic phase with *Im*3̄*m* space group symmetry, accompanied by an enlarged unit cell parameter of 141.9 Å (Figure 3G). This structural change was consistent with acid‐triggered electrostatic unlocking: protonation of OA weakens peptide–lipid anchoring, whereas increased protonation of VDAP enhances local cationic repulsion and interfacial hydration. These coupled interfacial changes drive structural relaxation toward a highly interconnected 3D aqueous channel network.

PLM observations provided complementary evidence for this graded phase transition. At pH 7.4, both Blank LLC and VDAP‐LLC exhibited birefringent textures characteristic of the anisotropic *H*_II_‐type mesophases (Figure 3H,I). At pH 6.5, VDAP‐LLC showed attenuated yet detectable birefringent textures, indicating residual anisotropic organization rather than a fully developed optically isotropic cubic state (Figure 3J). In contrast, no birefringent textures were observed at pH 5.0, yielding an optically isotropic dark field consistent with cubic phase formation (Figure 3K). To capture the dynamic optical response after acidification, time‐resolved PLM imaging was performed following medium exchange from pH 7.4 to pH 5.0. The initially strong birefringent textures progressively fragmented and weakened at 30 min, 1 h, and 6 h, and the field became predominantly optically isotropic at 24 h, with only weak residual birefringent textures (Figure S3). These dynamic optical changes support time‐dependent relaxation of the anisotropic *H*_II_‐type organization under acidic conditions.

To determine whether the pH‐dependent topology remodeling observed in VDAP‐LLC resulted from peptide incorporation, semaglutide was incorporated into the same LLC/OA matrix as a representative non‐cationic peptide comparator and analyzed by SAXS at pH 7.4, 6.5, and 5.0 (Figure S4). Semaglutide‐loaded LLC failed to undergo the pronounced pH‐triggered phase transitions exhibited by VDAP‐LLC. Instead, ordered mesophase features persisted across the tested pH range, with only minor structural changes upon acidification.

Overall, the surface charge, SAXS, and PLM results support a coupled electrostatic–topological gating mechanism. Interfacial VDAP‐OA electrostatic pairing stabilizes a compact *H*_II_ topology at physiological pH 7.4, whereas acidification progressively weakens this locked state, generating a mixed *H*_II_/cubic‐like intermediate at pH 6.5, and promoting conversion toward a bicontinuous *Im*3̄*m* network at pH 5.0. The semaglutide comparison further indicates that this pronounced topology remodeling is not a generic consequence of peptide incorporation into the LLC/OA matrix and supports an important contribution of cationic VDAP–OA interactions. Nevertheless, since semaglutide and VDAP differ in multiple physicochemical properties, these results do not establish cationic charge as the sole determinant of the observed topological regulation.

### 2.2 pH‐Gated VDAP Release Through Coupled Electrostatic Unlocking and Topology Switching

Having established that acidification concurrently weakens VDAP–OA electrostatic anchoring and remodels the LLC topology, VDAP release under physiological and acidic conditions was next evaluated (Figure 4A). At pH 7.4, VDAP‐LLC exhibited highly suppressed release, with cumulative release remaining below 10% over 7 days. This low‐leakage profile was consistent with the coupled electrostatic and topological locked state. As confirmed by zeta potential and SAXS analyses, electrostatic pairing between cationic VDAP and deprotonated OA reinforces a compact *H*_II_‐type organization, while its non‐interconnected one‐dimensional aqueous channels are associated with reduced peptide diffusion.

**FIGURE 4:**
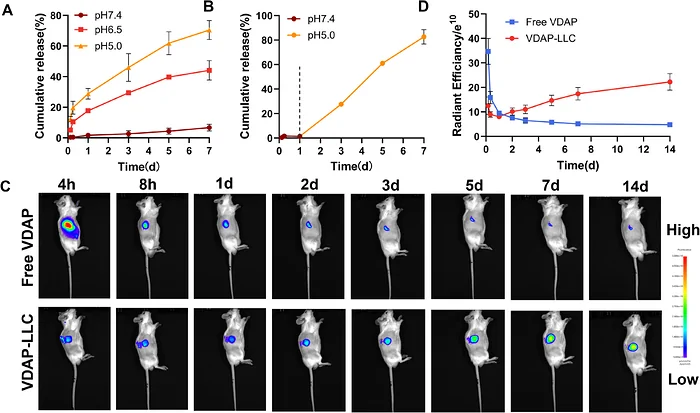
pH‐responsive VDAP release and in vivo depot retention. (A) Cumulative release profiles of VDAP from the structurally dynamic LLC depot in media at pH 7.4, 6.5, and 5.0 at 37°C. (B) Dynamic VDAP release in response to a stepwise pH challenge from pH 7.4 to pH 5.0. The vertical dashed line indicates the medium‐exchange time point. Data are presented as mean ± SD (*n* = 3). (C) Representative longitudinal fluorescence images of ICR mice after subcutaneous flank administration of free Cy5.5‐labeled VDAP or Cy5.5‐labeled VDAP‐LLC. (D) Average radiant efficiency within a consistently defined region of interest at the injection site over time. Data are presented as mean ± SD (*n* = 5). The fluorescence signal is a relative optical measure of local residence of Cy5.5‐bearing species and is not a direct measure of intact VDAP concentration or depot integrity.

In contrast, acidic conditions promoted VDAP release in a pH‐dependent manner. At pH 6.5, VDAP‐LLC showed an intermediate sustained‐release profile. This intermediate release behavior is associated with partial weakening of VDAP–OA electrostatic interactions, as suggested by the interfacial characterization, together with the intermediate structural organization revealed by SAXS and PLM. When the pH was further decreased to 5.0 (used to probe the maximal release response), the depot released more than 70% of the loaded VDAP. This accelerated release is consistent with a more complete electrostatic unlocking together with further remodeling from the compact *H*_II_ phase toward a more interconnected *Im*3̄*m* cubic network, collectively reducing matrix retention and diffusional restriction. To confirm that environmental pH dynamically regulates release activation rather than passive matrix degradation, a pH‐switch release experiment was performed (Figure 4B). During the initial incubation at pH 7.4, VDAP release remained limited. Upon switching the release medium to pH 5.0, the cumulative release curve showed a marked increase in release over the subsequent time points. This step‐change response demonstrates that VDAP‐LLC can translate external acidification into release activation, supporting its function as a pH‐gated electrostatic‐topological depot. Notably, as pH concurrently modulates electrostatic pairing and liquid‐crystalline topology, the data support a coupled electrostatic–topological gating mechanism rather than release control governed exclusively by phase behavior.

To assess whether pH‐gated release is uniquely mediated by specific peptide–lipid interactions, semaglutide release from the same LLC/OA carrier was monitored under identical conditions. Semaglutide‐loaded LLC exhibited relatively high cumulative released fractions at pH 7.4, 6.5, and 5.0, with only modest enhancement upon acidification (Figure S5). Thus, it did not reproduce the pronounced acidification‐associated retention‐to‐release transition observed with VDAP‐LLC. Taken together, the pH‐dependent VDAP release profiles, the dynamic pH‐switch response, and the semaglutide comparison support a coupled electrostatic–topological gating mechanism, whereby acidification converts VDAP‐LLC from a restrictive locked state into a more interconnected, transport‐favorable state.

Next, we performed in vivo evaluation of the formulation, employing Cy5.5 fluorescence as an optical proxy for the local residence behavior of Cy5.5‐bearing species originating from labeled VDAP after administration (Figure 4C). The local fluorescence signal of free Cy5.5‐labeled VDAP decreased rapidly, with an approximately 73% reduction within the first 24 h, consistent with rapid loss of fluorescent species from the injection site (Figure 4D). In contrast, VDAP‐LLC exhibited a localized fluorescence signal that persisted for more than 14 days, reaching an endpoint intensity 4.7‐fold higher than that of free VDAP.

Notably, the initial fluorescence intensity of VDAP‐LLC was lower than that of free VDAP. This difference may be related to concentration‐dependent self‐quenching of densely confined Cy5.5 labels within the condensed LLC matrix [55, 56], whereas interfacial hydration and spatial dilution may contribute to partial fluorescence recovery over time. Accordingly, fluorescence signal intensity should not be interpreted as a strict quantitative measure of active VDAP concentration. While Cy5.5‐based imaging delineates the spatial persistence of fluorophore‐tagged species, it lacks the specificity to distinguish intact Cy5.5‐VDAP from fluorescent cleavage products, nor does it provide direct evidence regarding the integrity of the underlying liquid‐crystalline matrix. Definitive assessment of intact‐peptide retention would require orthogonal validation, such as intact‐peptide LC–MS/MS or radiolabeling combined with chromatographic separation of intact peptide from labeled metabolites. Within these limitations, the sustained local fluorescence profile is consistent with prolonged residence of VDAP‐associated fluorescent species and reduced rapid loss from the injection region.

### 2.3 VDAP‐Mediated Tumor Cell Killing Elicits ICD‐Associated Danger Signaling

Having confirmed that VDAP‐LLC enables pH‐gated peptide release and prolonged local retention, the biological activity of the released peptide was subsequently evaluated. VDAP is a cationic, amphipathic α‐helical peptide that interacts with cellular membranes and induces membrane stress [57]. Beyond its role as a supramolecular regulator of the LLC matrix, this membrane‐active feature enables VDAP to trigger tumor cell death accompanied by immunogenic danger signaling. Therefore, the cytotoxicity and ICD‐associated DAMP emission of VDAP were evaluated in 4T1 cells, an aggressive triple‐negative breast cancer model.

VDAP exhibited dose‐ and time‐dependent cytotoxicity toward 4T1 cells, with an IC50 of 46.4 µM at 48 h (Figure 5A). Annexin V/PI flow cytometry further revealed a distinct cell death profile compared with doxorubicin (DOX), a classical ICD‐inducing chemotherapeutic control. At their respective effective concentrations, DOX (1 µM) predominantly induced late apoptosis after 48 h, whereas VDAP (100 µM) generated a mixed death pattern characterized by an apoptotic population of 34.3% and an increased PI‐positive population of 10.9% (Figure 5B,C and Figure S6). Given that Annexin V/PI staining primarily reflects phosphatidylserine exposure and membrane permeability, these data indicate that VDAP induces membrane‐disruptive tumor cell death rather than a specific regulated necrotic pathway. This death profile is consistent with the membrane‐active nature of VDAP and may facilitate extracellular release of immunostimulatory danger signals.

**FIGURE 5:**
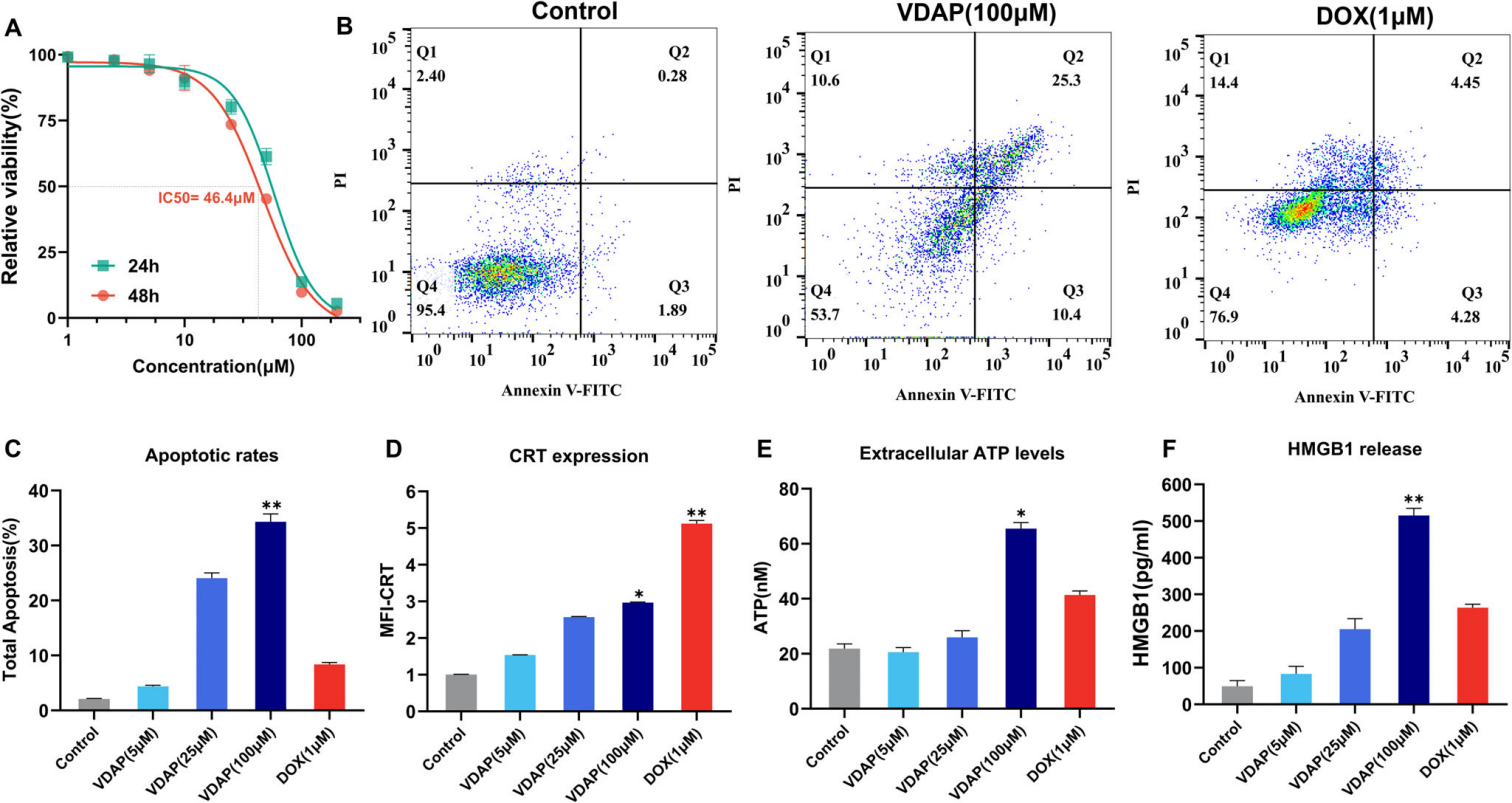
Cytotoxicity and ICD activity of VDAP in 4T1 cells. (A) Cell viability of 4T1 cells after treatment with different concentrations of VDAP for 24 or 48 h, determined by CCK‐8 assay (*n* = 4). (B) Representative flow cytometry plots of Annexin V‐FITC/PI staining after treatment with VDAP or DOX for 48 h. (C) Quantification of total apoptotic cells (Annexin V^+^) based on flow cytometry analysis. (D) Flow cytometric quantification of cell‐surface CRT exposure, expressed as mean fluorescence intensity (MFI) normalized to the untreated control group. (E) Extracellular ATP levels in culture supernatants detected by chemiluminescence assay. (F) Extracellular HMGB1 release quantified by ELISA. Data in (C–F) are presented as mean ± SD (*n* = 3). Statistical significance was determined using the Kruskal–Wallis test followed by Dunn's multiple comparisons test. *p < 0.05 and **p < 0.01 vs. untreated control cells.

We next assessed whether VDAP‐induced cytotoxicity was accompanied by hallmark ICD‐associated DAMP emission. Flow cytometry showed that VDAP promoted dose‐dependent calreticulin (CRT) exposure on the tumor cell surface (Figure 5D and Figure S7). At 100 µM, VDAP induced an approximately threefold increase in surface CRT compared with untreated controls, providing an “eat‐me” signal associated with phagocytic recognition. In parallel, VDAP triggered extracellular ATP release at levels comparable to the DOX‐treated group (Figure 5E), indicating the generation of a “find‐me” signal that can support dendritic cell recruitment and activation. HMGB1 secretion was also markedly increased after VDAP treatment, as confirmed by ELISA (Figure 5F). The elevated HMGB1 release was consistent with the increased membrane permeability observed in the Annexin V/PI assay and further supports the immunogenic nature of VDAP‐induced tumor cell death.

Taken together, these results demonstrate that VDAP induces tumor cell killing accompanied by ICD‐associated DAMP emission, including CRT exposure, ATP release, and HMGB1 secretion. Combined with the pH‐gated release profile of VDAP‐LLC, these results confirm the biological competence of the released peptide. Nevertheless, the present 2D models exhibit limited ability to capture the temporal interplay between topology‐regulated release and sustained DAMP emission, necessitating future validation in 3D postoperative tumor models containing intact depots under controlled pH environments.

### 2.4 Topology‐Switching VDAP‐LLC Suppresses Postoperative Recurrence and Pulmonary Metastasis

Having established that VDAP‐LLC enables pH‐gated peptide release and that VDAP elicits ICD‐associated danger signaling in vitro, we next evaluated whether this electrostatic‐topological depot could translate these mechanisms into therapeutic benefit in a postoperative tumor setting. An orthotopic 4T1 triple‐negative breast cancer model with incomplete surgical resection was established to mimic residual tumor disease after debulking surgery [58]. Approximately 90% of the primary tumor was removed, followed by localized administration of different formulations into the resection cavity according to the treatment schedule shown in Figure 6A.

**FIGURE 6:**
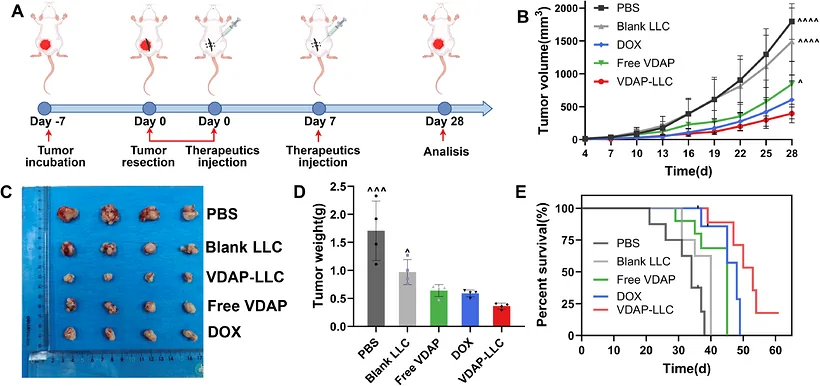
Postoperative antitumor efficacy of VDAP‐LLC against local recurrence. (A) Schematic timeline of the orthotopic 4T1 incomplete‐resection model and postoperative treatment schedule. All formulations were locally administered into the surgical bed on Day 0 immediately after incomplete resection and again on Day 7. Free DOX was administered at 5 mg kg^−^^1^ per administration as a local cytotoxic chemotherapy benchmark rather than as an optimized clinical regimen. (B) Tumor growth kinetics after postsurgical local treatment with PBS, Blank LLC, free VDAP, DOX, or VDAP‐LLC. Statistical comparisons were performed only for tumor volumes measured on Day 28. After assessment of normality using the Shapiro–Wilk test, Day 28 tumor volumes were analyzed using one‐way ANOVA followed by Dunnett's multiple comparisons test versus the VDAP‐LLC group. ^*p* < 0.05 and ^^^^*p* < 0.0001 vs. the VDAP‐LLC group. (C) Representative photographs of excised recurrent tumors collected from the prespecified terminal‐analysis cohort at the study endpoint Day 28. (D) Quantitative analysis of excised tumor weights from the prespecified terminal‐analysis cohort. Data are presented as mean ± SD (*n* = 4). Statistical significance was determined using the Kruskal–Wallis test followed by Dunn's multiple comparisons test. ^*p* < 0.05 and ^^^*p* < 0.001 vs. the VDAP‐LLC group. (E) Kaplan–Meier overall survival analysis during a 61‐day observation period(*n* = 8 mice per group at study initiation). Mice removed from the survival follow‐up for terminal tumor‐weight and downstream analyses were censored at the time of removal. Survival curves were compared using the Log‐rank Mantel–Cox test. The overall comparison showed significant differences among groups (*p* < 0.0001).

The residual tumor beds in the PBS and Blank LLC groups showed rapid local relapse, consistent with the aggressive recurrence pattern of the 4T1 model (Figure 6B). Free VDAP produced only transient tumor suppression, likely reflecting its limited local residence and rapid clearance from the postoperative tissue environment [59]. Free DOX showed a partial inhibition of postoperative recurrence under this predefined topical dosing schedule. As shown in Figure 6C,D, free DOX provided antirecurrence effects comparable to free VDAP; however, both remained inferior to VDAP‐LLC. In contrast, VDAP‐LLC markedly delayed postoperative tumor regrowth. At day 28, the excised recurrent tumors in the VDAP‐LLC group exhibited a mean mass of 0.36 g, corresponding to a tumor inhibition rate of 79% (Figure 6C,D). Compared to both free VDAP and free DOX, this enhanced anti‐recurrence efficacy demonstrates the importance of depot‐mediated peptide confinement and pH‐gated release. The therapeutic advantage of VDAP‐LLC further translated into prolonged survival. Mice treated with VDAP‐LLC exhibited a median survival of approximately 53 days, whereas control groups showed earlier mortality associated with rapid tumor progression (Figure 6E). These results collectively support the ability of VDAP‐LLC to prolong local VDAP activity and thereby improve postoperative tumor control.

Given that metastatic dissemination is a major cause of treatment failure in triple‐negative breast cancer, we next assessed whether localized VDAP‐LLC treatment could limit distant tumor spread. Longitudinal bioluminescence imaging revealed reduced systemic tumor burden in the VDAP‐LLC group compared with control treatments (Figure 7A,B). Histological analysis of lung tissues further confirmed this antimetastatic effect. Control mice showed extensive pulmonary metastatic lesions covering 39% of the lung cross‐sectional area. In contrast, VDAP‐LLC treatment preserved lung architecture and reduced metastatic burden to 4%, representing an approximately tenfold decrease (Figure 7C,D).

**FIGURE 7:**
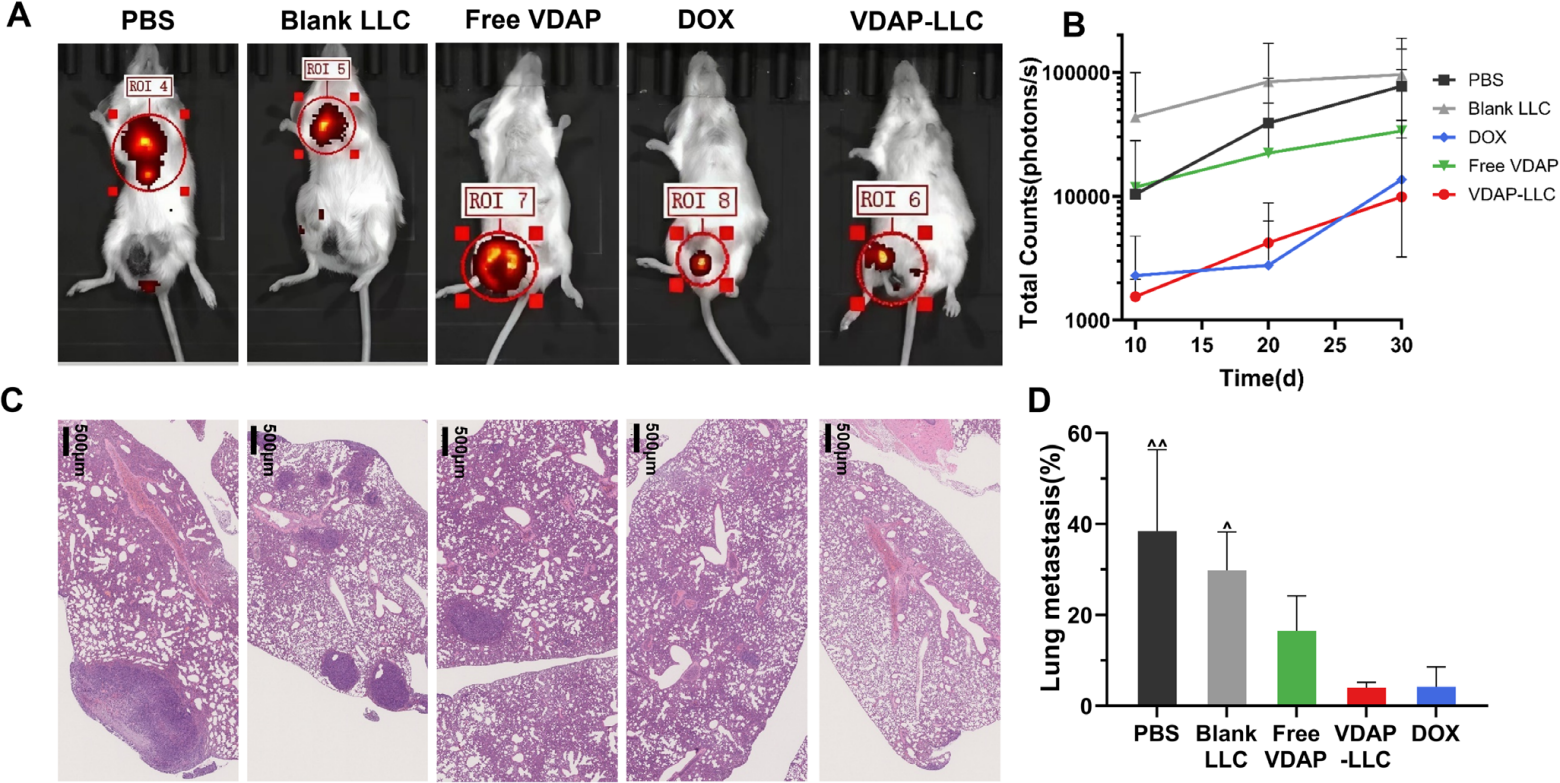
Suppression of systemic tumor burden and pulmonary metastasis by VDAP‐LLC. (A) Representative bioluminescence images of tumor‐bearing mice at the study endpoint, showing systemic tumor dissemination. (B) Longitudinal quantification of total photon flux reflecting viable tumor burden over time (*n* = 8). (C) Representative H&E staining of lung sections showing metastatic nodules (dense purple regions). Scale bar: 50 µm. (D) Quantitative analysis of metastatic area percentage relative to the total lung cross‐sectional area. Data are presented as mean ± SD (*n* = 3). Statistical significance was determined using the Kruskal–Wallis test followed by Dunn's multiple comparisons test. ^*p* < 0.05 and ^^*p* < 0.01 vs. the VDAP‐LLC group.

Taken together, these in vivo results demonstrate that local administration of VDAP‐LLC suppresses postoperative tumor recurrence and limits distant lung metastasis. Given that VDAP induces ICD‐associated DAMP emission in vitro, the systemic protection observed after localized depot therapy suggests the activation of antitumor immune mechanisms, which was further investigated through immune profiling in the following section.

### 2.5 VDAP‐LLC Remodels the Postsurgical Immune Microenvironment Through ICD‐Linked Antigen Presentation

To elucidate the immunological basis underlying the postoperative therapeutic efficacy, we examined whether topology‐programmed VDAP delivery could reshape the residual tumor bed into an immunogenic lesion. Given the ICD‐associated activity of VDAP observed in vitro, we first evaluated tumor cell damage and regulated cell death markers in vivo. Hematoxylin and eosin (H&E) staining showed expanded necrotic areas in VDAP‐LLC‐treated tumors, reflecting extensive tissue‐level destruction of residual tumor cells (Figure 8A). Consistently, VDAP‐LLC elevated p‐MLKL tissue positivity to 81% (vs. 71% for DOX), indicating enhanced necroptosis‐associated signaling (Figure 8B). Co‐immunofluorescence for Cleaved Caspase‐3 and TUNEL confirmed apoptosis‐associated DNA fragmentation, with the TUNEL‐positive area fraction increasing to 46%, compared to 29% in DOX‐treated controls (Figure 8C). These findings collectively suggest that sustained VDAP release drives multi‐modal tumor cell death displaying key ICD features.

**FIGURE 8:**
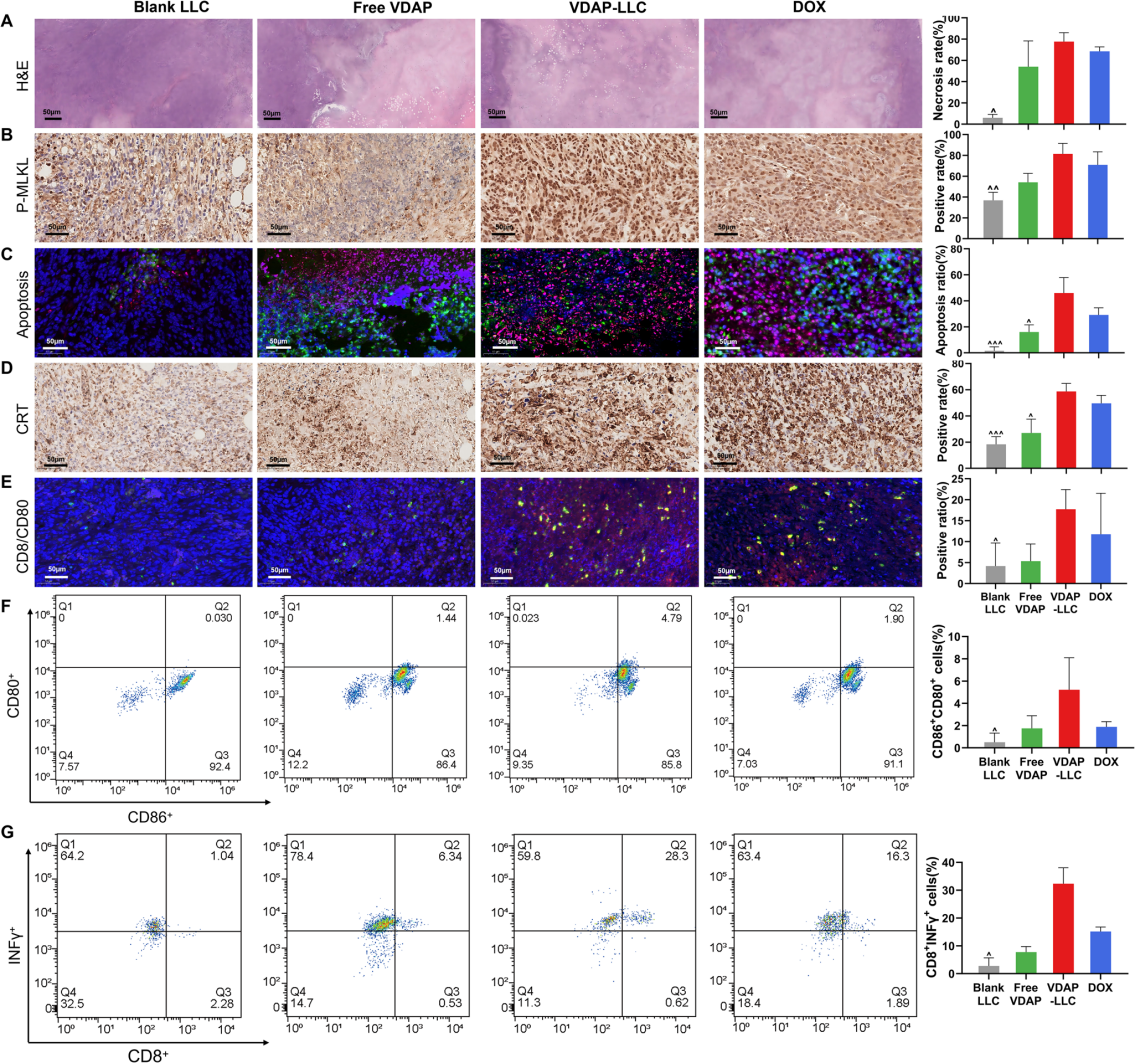
Local immune activation and systemic antitumor immunity induced by VDAP‐LLC. (A) Representative H&E staining of tumor sections and quantitative analysis of the necrotic area fraction. (B) Representative immunohistochemical staining and quantification of phosphorylated MLKL (p‐MLKL) tissue positivity. (C) Representative double immunofluorescence images of Cleaved Caspase‐3 (green) and TUNEL (red) in tumor tissues, with quantification of the TUNEL‐positive area fraction. (D) Representative immunohistochemical staining and quantification of CRT tissue positivity. (E) Immunofluorescence mapping of CD8^+^T lymphocytes (green) and CD80^+^antigen‐presenting cells (red) within the tumor microenvironment, with quantitative analysis of spatial colocalization. (F) Representative flow cytometry plots and quantification of systemic mature dendritic cells (CD80^+^CD86^+^) in the spleen, gated on CD45^+^CD11c^+^MHCII^+^ cells. (G) Representative flow cytometry plots and quantification of functional intratumoral cytotoxic T cells (CD8^+^IFN‐γ^+^), gated on CD3^+^ CD8^+^ T cells. Data are presented as mean ± SD (*n* = 3). Statistical significance was determined using the Kruskal–Wallis test followed by Dunn's multiple comparisons test. ^*p* < 0.05, ^^*p* < 0.01, and ^^^*p* < 0.001 vs. the VDAP‐LLC group. Scale bars: 50 µm.

We next examined whether this mixed tumor cell‐death pattern translated into ICD‐associated DAMP generation within the residual tumor bed. Immunohistochemical analysis showed that VDAP‐LLC markedly enhanced the expression of key DAMPs, including CRT, HMGB1, and HSP70, relative to control treatments (Figure 8D and Figure S8). Quantitatively, positive area fractions reached 59% for CRT, 66% for HMGB1, and 72% for HSP70. These DAMP signatures were consistent with the in vitro ICD profile of VDAP and support the formation of an immunogenic tumor bed, in which CRT exposure may facilitate phagocytic recognition and HMGB1 and HSP70 provide danger‐associated cues for antigen uptake and antigen‐presenting cell activation.

Consistent with this DAMP‐enriched microenvironment, VDAP‐LLC enhanced local immune cell remodeling within the residual tumor bed. Immunofluorescence analysis revealed increased infiltration of CD80^+^ antigen‐presenting cells and CD8^+^ T cells, with evident spatial proximity between these populations (Figure 8E). The CD80^+^ APC/ CD8^+^T cell co‐localization fraction reached 18%, indicating enhanced local interaction between antigen‐presenting cells and effector lymphocytes within the postoperative niche.

Flow cytometry further confirmed that local immune remodeling was accompanied by systemic antigen‐presenting activation and intratumoral cytotoxic T‐cell responses. In the spleen, VDAP‐LLC increased the abundance of mature dendritic cells, defined as CD80^+^CD86^+^ cells within the CD11c^+^MHC II^+^ population, to 5.2%, compared with 1.9% in the DOX group (Figure 8F; gating strategies are shown in Figure S9). Within the tumor, VDAP‐LLC markedly increased the proportion of IFN‐γ‐secreting CD8^+^ T cells among CD3^+^CD8^+^ T cells to 32.4%, representing a more than twofold increase over DOX (15.2%) and an 11‐fold increase over Blank LLC (2.8%) (Figure 8G).

Overall, these results demonstrate that VDAP‐LLC converts sustained local peptide delivery into an ICD‐linked antitumor immune cascade. The depot promotes tumor cell death and DAMP release in the residual tumor bed, enhances antigen‐presenting cell engagement, supports systemic DC maturation, and facilitates functional CD8^+^ T cell activation. This immune remodeling provides a mechanistic explanation for the suppression of postsurgical recurrence observed with VDAP‐LLC. Nevertheless, our current controls do not fully differentiate sequence‐dependent VDAP activity from nonspecific membrane perturbation. Future studies incorporating a physicochemically matched scrambled or non‐functional peptide control are warranted to determine the sequence dependence of these therapeutic and immunomodulatory effects. Mechanistically, VDAP acts as an ICD inducer that promotes endogenous antigen release and presentation rather than serving as a predefined target antigen [31]. However, our population‐level DC and T‐cell metrics do not prove whether activated CD8^+^ T cells specifically target 4T1‐derived antigens. Future studies using ex vivo restimulation with 4T1 antigen preparations, followed by IFN‐γ ELISPOT, intracellular cytokine staining, or cytotoxicity assays, are required to confirm tumor‐specific reactivity and define the functional breadth of the T‐cell response.

### 2.6 Topology‐Programmed Local Delivery Improves the Therapeutic Window

Given that VDAP is a membrane‐active peptide with potent tumor‐killing activity, an essential requirement for its postoperative application is to confine its cytotoxic effect to the residual tumor bed while minimizing systemic exposure. The pH‐gated electrostatic‐topological design of VDAP‐LLC was intended to address this challenge by maintaining low basal leakage under physiological conditions and enabling localized release in acidic tumor‐associated environments. We therefore evaluated the systemic biosafety of VDAP‐LLC by histological examination of major organs, hematological analysis, and serum biochemical profiling.

Histological analysis showed no marked inflammatory lesions, necrosis, or structural abnormalities in major organs of VDAP‐LLC‐treated mice (Figure 9A), indicating that local depot administration did not cause marked organ systemic organ toxicity.

**FIGURE 9:**
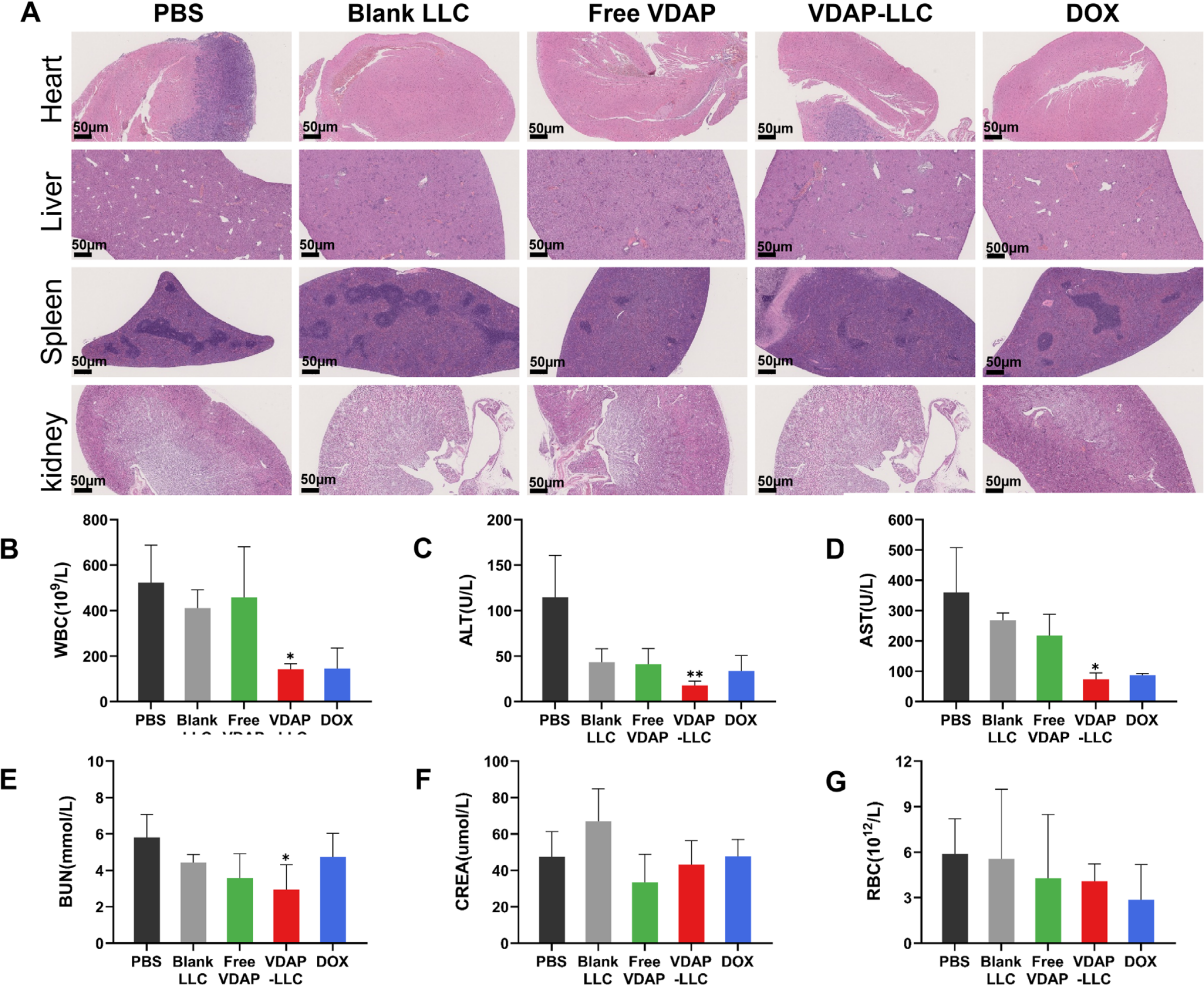
Systemic biosafety and biocompatibility of VDAP‐LLC. (A) Representative H&E staining of major organs (heart, liver, spleen, and kidney) collected from mice in different treatment groups. (B) White blood cell (WBC) counts in peripheral blood. (C, D) Serum levels of liver function markers: (C) aspartate aminotransferase (AST) and (D) alanine aminotransferase (ALT). (E, F) Serum levels of kidney function markers: (E) blood urea nitrogen (BUN) and (F) creatinine (CREA). (G) Red blood cell (RBC) counts. Data are presented as mean±SD (*n* = 3). Statistical significance was determined using the Kruskal–Wallis test followed by Dunn's multiple comparisons test. *p < 0.05 and **p < 0.01 vs. the PBS group.

Hematological and biochemical analyses further revealed that local VDAP‐LLC treatment did not exacerbate systemic toxicity. In the PBS‐treated group, rapid tumor progression was accompanied by marked systemic abnormalities, including elevation of white blood cell counts and increased serum AST and ALT levels, consistent with tumor‐burden‐associated inflammation and liver stress (Figure 9B–D). In contrast, VDAP‐LLC treatment reduced WBC counts and attenuated AST and ALT elevations, consistent with its effective suppression of tumor recurrence. Renal function markers, including BUN and CREA, remained stable after VDAP‐LLC treatment (Figure 9E,F), suggesting an absence of renal toxicity.

Importantly, VDAP‐LLC circumvented the hematological toxicity inherent to local free DOX administration. Although DOX partially reduced tumor‐associated systemic stress through tumor suppression, it decreased red blood cell counts, indicating chemotherapy‐associated myelosuppression (Figure 9G). Conversely, VDAP‐LLC maintained hematological homeostasis while achieving strong antitumor efficacy. These results demonstrate that topology‐programmed local delivery can balance sustained postoperative tumor control with systemic host protection, thereby expanding the therapeutic window of VDAP‐LLC for localized cancer immunotherapy.

Collectively, these findings establish an immunological link between topology‐programmed VDAP release and postoperative antitumor efficacy. Sustained peptide delivery from the LLC depot induced mixed tumor cell damage with ICD‐compatible features, enhanced DAMP signaling, promoted antigen‐presenting cell engagement, and supported systemic DC maturation and CD8^+^T cell activation. This local‐to‐systemic immune cascade provides a mechanistic basis for the observed suppression of residual tumor regrowth and pulmonary metastasis in the postsurgical TNBC model.

Although the 4T1 TNBC model provides a relevant, immunocompetent setting for proof‐of‐concept evaluation, the present in vivo validation was limited to a single tumor model. Future studies employing additional immunologically distinct, immunocompetent tumor models are needed to determine the generalizability of VDAP‐LLC across different tumor immune microenvironments.

## 3 Conclusions

In summary, we engineered an adaptive LLC depot regulated by a therapeutic peptide, where the cationic VDAP‐protonatable OA electrostatic coupling acts as an electrostatic‐topology gate. Under physiological conditions, this cooperative electrostatic assembly stabilizes a compact *H*_II_ phase and minimizes premature peptide leakage. Microenvironmental acidification weakens this electrostatic lock and promotes graded topology remodeling toward a bicontinuous *Im*3̄*m* cubic network, thereby coupling structural relaxation with pH‐gated VDAP release. The released peptide induces ICD‐associated tumor cell damage, thereby promoting DAMP‐mediated antigen presentation, functional CD8^+^ T‐cell responses, and resulting in postoperative immune remodeling. In a postsurgical 4T1 TNBC model, this VDAP‐LLC depot suppressed residual tumor regrowth, reduced pulmonary metastasis, prolonged survival, and showed favorable systemic biosafety under the tested regimen. Within the demonstrated depot platform, these results support that pH‐responsive, amphipathic peptide–lipid interfacial features can simultaneously drive supramolecular assembly, control drug release, and orchestrate local immunity; whether other cationic peptides can achieve comparable topological locking remains to be determined on a case‐by‐case basis.

## 4 Experimental Section

### 4.1 Materials and Cell Lines

Soybean phospholipid (SPC) was purchased from Lipoid GmbH (Germany). Sorbitan monooleate (SMO) and oleic acid (OA) were provided by Croda. The Vpr‐derived antitumor peptide (VDAP, sequence: IIRILQQLLFIHFRIGCRHSRIGVTRQRRARNG, purity > 95%) was synthesized by Zhejiang Peptide Biotechnology Co., Ltd. (China). Doxorubicin was obtained from Shenzhen Main Luck Pharmaceuticals Inc. (China).

Analytical kits, including the Cell Counting Kit‐8 (Cat. No. IV08‐100, Invigentech), Annexin V‐FITC Apoptosis Detection Kit (Cat. No. C1062S, Beyotime), ATP Assay Kit (Cat. No. MA0440, Meilunbio), Mouse Acetyl‐HMGB1 ELISA Kit (Cat. No. ml107157, Mlbio), and CF488 TUNEL Apoptosis Kit (Cat. No. G1504‐50T, Servicebio), were acquired and utilized according to the manufacturers' protocols.

For histological and immunofluorescence evaluations, primary antibodies including anti‐Calreticulin (ET1608‐60), anti‐HMGB1 (ET1601‐2), anti‐HSP70 (ET1601‐11), and anti‐p‐MLKL (ET1705‐51) were purchased from Huabiao (China). Anti‐CD8 (YA6867) and anti‐CD80 (YA3386) were purchased from MedChemExpress, and anti‐cleaved caspase‐3 (GB115733) from Servicebio. The YSFluor 594‐conjugated secondary antibody (33112ES60) was obtained from Yeasen (China).

For flow cytometry assays, all fluorochrome‐conjugated antibodies were globally sourced from Proteintech (China), including anti‐CD45 (98035‐1‐RR), anti‐CD8 (98472‐2‐RR), anti‐CD3 (AY7‐FcA65659), anti‐IFN‐γ (Cat. No. 98045‐1‐RR), anti‐CD11c (65130‐1‐Ig), anti‐CD80 (98158‐1‐RR), and anti‐CD86 (APC‐FcA98025). All other chemical reagents were of analytical grade.

The murine TNBC cell line 4T1 was obtained from Procell (China). Luciferase‐expressing 4T1 cells (4T1‐Luc, Cat. No. IML‐021) were purchased from Yimo Biotech (China). Both cell lines were cultured in RPMI‐1640 medium supplemented with 10% fetal bovine serum (FBS) and 1% penicillin/streptomycin, and maintained at 37°C in a humidified 5% CO_2_ atmosphere. Murine 4T1 triple‐negative breast cancer cells (female BALB/c origin) were maintained at low passage (≤ 20) for all experiments.

### 4.2 Bioinformatics Analysis and In Vitro Cellular Assays

#### 4.2.1 Bioinformatics

The physicochemical properties and structural topology of the VDAP peptide were analyzed using web‐based bioinformatics tools. The theoretical net charge and isoelectric point (pI) were calculated using the PepCalc server (Innovagen AB, https://pepcalc.com/). Furthermore, the amphipathic *α*‐helical structure, specifically the segregation of hydrophobic and hydrophilic faces, was visualized using the NetWheels application (http://www.lbqp.unb.br/NetWheels/).

#### 4.2.2 Cytotoxicity and Apoptosis

To evaluate cytotoxicity, 4T1 cells were seeded into 96‐well plates (6×10^3^ cells/well). Following overnight adherence, cells were treated with varying concentrations of VDAP (1–200 µM) for 24 and 48 h. Cell viability was quantified via the CCK‐8 assay (absorbance at 450 nm; SPARK 10 M, Tecan). For apoptosis analysis, cells were seeded in 6‐well plates (4×10^5^ cells/well) and exposed to designated concentrations of VDAP (5, 25, and 100 µM) or DOX (1 µM, serving as a positive control) for 48 h. Treated cells were harvested utilizing an enzyme‐free cell dissociation buffer to preserve phosphatidylserine‐calcium interactions, stained with the Annexin V‐FITC/PI Detection Kit, and analyzed via flow cytometry (Guava easyCyte, Luminex).

#### 4.2.3 ICD Assessment

To assess the early immunogenic translocation of CRT, 4T1 cells (4×10^5^ cells/well) were treated with VDAP or DOX for 16 h. Non‐permeabilized cells were blocked with 2% BSA on ice for 30 min to prevent non‐specific binding, subsequently incubated with an anti‐CRT primary antibody (1:100) overnight at 4°C, and labeled with a YSFluor 594‐conjugated secondary antibody (1:200) for 1 h in the dark prior to flow cytometric analysis. To quantify late‐stage DAMP release, cells were treated for 48 h. Culture supernatants were harvested and centrifuged (2000 rpm, 5 min) to eliminate cellular debris. Extracellular ATP and HMGB1 levels were subsequently determined using their respective assay kits following the manufacturers' protocols.

### 4.3 Preparation and Macroscopic Validation of Structurally Dynamic Depots

The basal lipid precursor was prepared by co‐dissolving SPC, SMO, ethanol, and DMSO at a precise relative mass ratio of 35:35:10:10. To construct the pH‐responsive gating system, the molecular switch (OA) was incorporated into the basal matrix at designated mass fractions (0, 2, 5, and 10 wt.%, calculated as *W_0A_*/(*W_basal_* +*W_OA_*)×100%), corresponding to formulations F0, F1, F2, and F3. Mixtures were agitated at 40 rpm (25°C) for 12 h to achieve optical isotropy. F2 (5 wt.% OA) was subsequently identified as the optimal structural matrix for downstream applications (hereafter denoted as the Blank LLC).

For payload integration, VDAP was molecularly dispersed in DMSO via mild bath sonication and homogeneously blended into the precursor to yield a final peptide concentration of 10 mg g^−1^ (hereafter denoted as the VDAP‐LLC). For the non‐cationic peptide control, VDAP was replaced with semaglutide while maintaining the same LLC/OA composition and peptide loading concentration; the resulting formulation was designated semaglutide‐loaded LLC.

The in situ sol‐to‐gel phase transition and clinical injectability were macroscopically validated by extruding the precursors (100 µL) into an aqueous PBS bath (5 mL, pH 7.4, 37°C) utilizing a standard 26G needle. Structural fidelity of the dynamically formed depots was evaluated after an 8 h equilibration period; only formulations that maintained a cohesive three‐dimensional architecture without macroscopic disintegration or phase separation were classified as stable and selected for subsequent characterizations.

### 4.4 Physicochemical and Structural Characterization

#### 4.4.1 Rheology

Rheological properties were characterized utilizing an MCR 102 modular compact rheometer (Anton Paar, Austria) equipped with a 25 mm parallel plate geometry (0.5 mm gap). To evaluate injectability, steady‐state shear measurements of the liquid precursors were first performed at 25°C by ramping the shear rate from 10 to 100 s^−^^1^. Subsequently, to evaluate the mechanical rigidity of the formed gel, the dynamic depots were equilibrated in respective buffers (pH 7.4 or 5.0) for 24 h at 37°C. Strain amplitude sweeps were conducted by ramping the shear strain(γ) from 0.01% to 100% at a fixed frequency of 1 Hz. The plateau *G'*, utilized to quantify matrix rigidity, was extracted from the linear viscoelastic (LVE) region of the amplitude sweep curves.

#### 4.4.2 Zeta Potential Analysis

Since bulk highly viscous liquid crystalline phases are unsuitable for electrophoretic light scattering, dispersed structural surrogates (cubosomes/hexosomes) were generated. Briefly, 50 mg of the precursor was dispersed into 5 mL of defined 10 mM low‐ionic‐strength buffers to maintain measurement stability: HEPES (pH 7.4), MES (pH 6.5), or acetate buffer (pH 5.0). Dispersions were homogenized utilizing a probe sonicator (30% amplitude, 30 s on / 30 s off cycle, for 2 min total sonication time). This protocol was performed in an ice bath to prevent any thermal degradation of the lipid matrices and the loaded peptide. The Zeta potential was determined utilizing a Zetasizer Nano ZS (Malvern Instruments, UK) at 25°C via the Smoluchowski approximation.

#### 4.4.3 Topological Phase Determination

Mesophase textures were directly observed at 37°C using polarized light microscopy (PLM; WMP‐6503, China). For endpoint PLM analysis, gel samples equilibrated under the indicated pH conditions were sandwiched between a glass slide and coverslip before imaging. To monitor the dynamic optical response after acidification, time‐resolved PLM imaging was performed following medium exchange from pH 7.4 to pH 5.0. Images were acquired at 0 min, 30 min, 1 h, 6 h, and 24 h after pH adjustment. The nanoscale mesophase organization was analyzed by SAXS using a Xeuss 3.0 C system (Xenocs, France) equipped with a Cu Kα source(λ = 0.154 nm). Gel samples equilibrated at the indicated pH values were sealed between Kapton films. Scattering profiles were acquired at 37°C with a sample‐to‐detector distance of 575 mm and an exposure time of 10 min, followed by azimuthal averaging to obtain 1D profiles. For both Blank‐LLC and VDAP‐LLC at pH 7.4, Bragg peaks were indexed to the inverse hexagonal *H*_II_ (spatial ratio 1: √3: 2), with the lattice parameter calculated as *a_hex_* = 4π ∕ (√3*q*_10_). For VDAP‐LLC at pH 6.5, the first three peak‐position ratios (1, 1.690, and 1.940) were close to the characteristic *H*_II_ reflections, whereas additional higher‐*q* features (2.333, 2.940, and 3.833) approximated Im3̄m‐related reflections. This pattern was therefore assigned to a mixed *H*_II_/cubic‐like intermediate state rather than a complete phase transition. Upon acidification to pH 5.0, the VDAP‐LLC exhibited a transition toward the primitive bicontinuous cubic *Im*3̄*m* phase, confirmed by the characteristic reflection sequence (√2: √6: √8: √12), where the theoretically allowed (200) reflection (corresponding to√4) is characteristically suppressed by the bilayer form factor minimum. The lattice parameter for the cubic phase was subsequently derived as *a_cub_* = 2π√(*h* ^2^+*k* ^2^+*l* ^2^)∕ *q_hkl_*. Semaglutide‐loaded LLC was analyzed under the same SAXS conditions at pH 7.4, 6.5, and 5.0 to provide a non‐cationic peptide control for comparison with VDAP‐LLC.

### 4.5 In Vitro Release Kinetics and In Vivo Retention

#### 4.5.1 Release Kinetics

The VDAP‐LLC precursor (0.1 mL) was injected into 10 mL of pre‐warmed release media at 37°C to establish strict sink conditions. To evaluate pH‐dependent release profiles without compromising buffering capacity, three buffering systems were utilized: PBS (pH 7.4), MES buffer (pH 6.5), and acetate buffer (pH 5.0). Depots were maintained under constant orbital agitation (100 rpm). At predetermined intervals, 5 mL aliquots were withdrawn and immediately replenished with an equal volume of the respective fresh buffer. For the step‐wise pH challenge (pH‐switch experiment), depots were initially incubated in pH 7.4 PBS for 24 h, followed by a complete medium replacement with pH 5.0 acetate buffer. VDAP concentrations were quantified via HPLC (Ultimate 3000, Thermo Fisher Scientific, USA) utilizing an Agilent ZORBAX 300SB‐C18 column (4.6 × 150 mm, 3.5 µm) maintained at 30°C. The isocratic mobile phase consisted of acetonitrile/water containing 0.01% TFA (33:67, v/v) at a flow rate of 1.0 mL min^−1^with UV detection at 220 nm. Semaglutide‐loaded LLC was evaluated in parallel under the same release conditions, including the same depot mass, release‐medium volume, buffer composition, temperature, orbital agitation, sampling schedule, and medium‐replacement procedure. VDAP and semaglutide concentrations in the collected release media were quantified by HPLC using analyte‐specific calibration curves under the corresponding chromatographic conditions.

#### 4.5.2 In Vivo Fluorescence Retention Tracking

To enable longitudinal near‐infrared fluorescence tracking, VDAP was covalently conjugated with Cy5.5‐SE at a feed molar ratio of 1:1 and purified by exhaustive dialysis (MWCO 2000 Da) against deionized water for 24 h to minimize residual unreacted dye. Male ICR mice (6–8 weeks, *n* = 5) were depilated and received a subcutaneous flank injection of either the free Cy5.5‐labeled VDAP solution or Cy5.5‐labeled VDAP‐LLC (100 µL). Local fluorescence was monitored for 14 days using an AniView 600 live animal imaging system (BLT, China). After background subtraction, radiant efficiency was quantified within a consistently defined region of interest centered on the injection site. The fluorescence signal served as a relative optical indicator of the local residence of Cy5.5‐bearing species and was not used as a direct measure of intact VDAP concentration or depot integrity.

### 4.6 Model of Simulated Incomplete Tumor Resection and In Vivo Antitumor Efficacy

All in vivo animal experiments were commissioned to and conducted at the animal facility of Dr. Can Biotechnology (Zhejiang) Co., Ltd. (China). The animal care and experimental protocols were approved by the Institutional Animal Care and Use Committee (IACUC) of Dr. Can Biotechnology (Zhejiang) Co., Ltd. (Protocol No. DRK‐202507019854). Female BALB/c mice (6–8 weeks, 18–20 g) were housed under specific pathogen‐free (SPF) conditions. To establish the clinically relevant incomplete resection model, 4T1‐Luc cells (4 × 10^6^) were orthotopically injected into the fourth mammary fat pad. Upon primary tumors reaching approximately 100 mm^3^(typically on Day 7), approximately 90% of the macroscopic tumor mass was surgically resected. A microscopic tumor remnant was deliberately preserved to simulate clinically positive margins, followed by surgical suturing of the incision. After surgery, the mice were randomly divided into five groups (*n* = 8 per group): PBS, Blank‐LLC, Free VDAP (15 mg kg^−^^1^), Free DOX (5 mg kg^−^^1^) and VDAP‐LLC (15 mg kg^−^^1^ VDAP equivalent). Each formulation was locally administered into the surgical bed immediately after incomplete resection on Day 0 and again on Day 7 (Figure 6A). Free DOX was administered locally as a cytotoxic chemotherapy benchmark to mirror the local postoperative schedule rather than as an optimized systemic or clinical multi‐cycle regimen. Tumor recurrence was monitored every 3 days using digital calipers, and the recurrent tumor volume was calculated as *V* = (Length × Width^2^) / 2. At the experimental endpoint for tumor growth analysis (Day 28), the recurrent tumors were excised, weighed, and photographed. To evaluate overall survival, an independent animal cohort was monitored continuously for 61 days, with euthanasia performed upon signs of severe distress or excessive tumor burden in compliance with ethical guidelines.

Longitudinal whole‐body tumor burden and pulmonary metastasis were evaluated using an IVIS Lumina XRMS system (PerkinElmer). Imaging was performed under isoflurane anesthesia 10 min post‐intraperitoneal injection of D‐Luciferin (150 mg kg^−1^). Bioluminescence signals were quantified using Living Image software within standardized ROI covering the tumor site or whole body, and data were expressed as Total Counts (photons s^−1^).

### 4.7 Histological, Immunofluorescence, and Flow Cytometric Analyses

#### 4.7.1 Tissue Sectioning and Staining

Excised tumors and major organs (harvested on Day 28) were fixed in 10% neutral buffered formalin, paraffin‐embedded, and sectioned at 4 µm thickness. Sections underwent standard H&E staining for toxicity evaluation and necrosis quantification. For immunohistochemistry, following antigen retrieval in EDTA buffer (pH 9.0), sections were blocked and incubated overnight with primary antibodies against CRT, HMGB1, HSP70, and p‐MLKL, followed by standard HRP‐DAB detection and hematoxylin counterstaining. Apoptosis and immune infiltration were evaluated using a combinatorial TUNEL (Alexa Fluor 488)/Cleaved Caspase‐3 (Alexa Fluor 647) IF assay (with DAPI counterstain) and CD8/CD80 IF staining (using FITC‐ and Cy3‐conjugated secondary antibodies), respectively. Slides were digitized via a 3DHistech Pannoramic Midi scanner, and marker expression (quantified as the positive area fraction relative to total tissue area) was segmented and processed using ImageJ software with consistent intensity thresholds across all samples.

#### 4.7.2 Flow Cytometry

Tumors and spleens were harvested on Day 28 for immune profiling. Tumors were enzymatically digested with DNase I (100 U mL^−1^) and Collagenase D (1 mg mL^−1^) for 1–2 h at 37°C, and then subjected to Percoll gradient centrifugation to enrich tumor‐infiltrating lymphocytes (TILs). Spleens were mechanically dispersed into single‐cell suspensions. The single‐cell suspensions were blocked and stained with fluorochrome‐conjugated antibodies against surface markers (CD45, CD3, CD8, CD11c, CD80, and CD86). For intracellular IFN‐γ detection, cells were pre‐incubated in a serum‐free medium containing a protein transport inhibitor Brefeldin A for 4–6 h to completely block cellular cytokine secretion. Subsequently, cells were surface‐stained, fixed, permeabilized, and stained intracellularly. Data were acquired on an Agilent NovoCyte Opteon cytometer and analyzed using FlowJo software. The specific gating strategies focused on activated cytotoxic T lymphocytes (CD3^+^CD8^+^IFN‐γ^+^) and mature DC (CD11c^+^CD80^+^CD86^+^).

### 4.8 Systemic Toxicity and Statistical Analysis

#### 4.8.1 Systemic Toxicity Assessment

On Day 28, blood samples were collected via orbital sinus puncture. Whole blood was directly utilized to evaluate hematological parameters (WBC, RBC, HGB). Concurrently, serum was carefully isolated via centrifugation (2000 g, 10 min) and subjected to biochemical analysis of hepatic (ALT, AST) and renal (BUN, CREA) function markers using an automated biochemical analyzer.

#### 4.8.2 Statistical Analysis

All quantitative data were presented as mean ± SD unless otherwise stated. The sample size (*n*) for each experiment is indicated in the corresponding figure legends, with *n* ≥ 3 independent biological or experimental replicates unless otherwise specified. No data transformation was performed, and no data points were excluded from statistical analysis, unless explicitly stated. Normalization was performed only where specified in the corresponding figure legend, such as CRT mean fluorescence intensity normalized to that of the untreated control group. Data normality was assessed using the Shapiro–Wilk test when applicable. Normally distributed data involving multiple‐group were analyzed using one‐way ANOVA followed by Tukey's or Dunnett's multiple comparisons test, as specified in the corresponding figure legends. For the tumor growth experiment, longitudinal growth curves were presented descriptively, and statistical comparisons were restricted to tumor volumes measured on Day 28, which were analyzed using one‐way ANOVA followed by Dunnett's multiple comparisons test, with the VDAP‐LLC group as the reference. When data failed normality testing, or sample sizes were insufficient to evaluate distribution, multiple‐group comparisons were performed using the Kruskal–Wallis test followed by Dunn's multiple comparisons test. Pairwise comparisons between Blank LLC and VDAP‐LLC at each pH value in the zeta‐potential analysis were performed using Mann–Whitney tests with correction for multiple comparisons. Survival curves were generated using the Kaplan–Meier method and compared using the Log‐rank Mantel–Cox test. Mice removed from survival follow‐up for prespecified terminal tumor‐weight and downstream analyses were censored at the time of removal. All statistical tests were two‐sided unless otherwise stated and were performed using GraphPad Prism 8.0. In graphical representations, *, **, ***, and **** denote *p* < 0.05, *p* < 0.01, *p* < 0.001, and *p* < 0.0001, respectively, compared with the control group explicitly identified in the corresponding figure legend; ^, ^^, ^^^, and ^^^^ denote *p* < 0.05, *p* < 0.01, *p* < 0.001, and *p* < 0.0001, respectively, compared with the VDAP‐LLC group, unless otherwise indicated. A *p* value < 0.05 was considered statistically significant

### 4.9 Declaration of Generative AI and AI‐Assisted Technologies in the Writing Process

During the preparation of this manuscript, the authors used Gemini 3.1 Pro, an AI‐based natural language processing tool (large language model), to polish the English language, refine the logical flow of specific text segments, and assist with academic formatting guidelines. The technology was utilized for language and structural editing purposes, and no AI tools were employed to generate original data, alter scientific results, or formulate scientific conclusions. After using this tool/service, the authors reviewed and edited all the AI‐generated suggestions. The authors take full and ultimate responsibility for the final content, data integrity, and scientific conclusions of this publication.

## Author Contributions

**Yan Yan**: conceptualization, methodology, investigation, formal analysis, data curation, visualization, writing – original draft, writing – review and editing. **Bin Chen**: methodology, resources, validation. **Yanqing Wang**: methodology, resources, validation. **Yuxi Hou**: investigation, data curation, validation. **Zhenlin Ma**: investigation, validation, data curation. **Zini Yang**: investigation, resources. **Xia Yin**: writing – review and editing, validation. **Yiling Chen**: validation, writing – review and editing. **Litao Huang**: validation, writing – review and editing. **Menghan Cai**: validation, writing – review and editing. **Xinlin Liu**: writing – review and editing, formal analysis. **Yi Zhun Zhu**: writing – review and editing, formal analysis. **Li Ye**: conceptualization, methodology, funding acquisition, project administration, supervision, writing – review and editing, validation.

## Funding

This work was supported by the Macao Science and Technology Development Fund (No. FDCT 0019/2024/RIA1, 0148/2022/A3 and 0013/2026/RIB1).

## Conflicts of Interest

The authors declare no conflicts of interest.

## Supporting information




**Supporting File**: advs78360‐sup‐0001‐SuppMat.docx.

## Data Availability

The data that support the findings of this study are available on request from the corresponding author. The data are not publicly available due to privacy or ethical restrictions.
