# Supplementary material for: Therapeutic Peptide‐Programmed Liquid‐Crystalline Topology Switching for pH‐Gated Postoperative Cancer Immunotherapy
Source: Adv Sci (Weinh). 2026 Oct 18:e78360. Online ahead of print. doi: 10.1002/advs.78360 (PMC13676987; doi:10.1002/advs.78360)
Supplement: Supplementary file 1 [file ADVS-9999-e78360-s001.docx]

Supporting Information

**Therapeutic Peptide-Programmed Liquid-Crystalline Topology Switching for pH-Gated Postoperative Cancer Immunotherapy**

*Yan Yan^1,2^, Bin Chen^2^, Yanqing Wang^2^, Yuxi Hou^1^, Zhenlin Ma^1^, Zini Yang^2^, Xia Yin^1,2^, Yiling Chen^1^, Litao Huang^1^,* *Menghan Cai^1^, Xinlin Liu^1,3^, Yi Zhun Zhu^1^, Li Ye^1*^*

**Table S1. Composition and screening parameters of the lipid liquid crystal precursors.**

| **Code** | **OA Content (wt%)^a^** | **Basal Phase Ratio^b^** | **Structural Integrity^c^** | ***G*'( kPa)^d^** | **Decision** |
| --- | --- | --- | --- | --- | --- |
| **F0** | 0% | 35 : 35 : 10 : 10 | Disintegrated | 0.1±0.04 | Excluded |
| **F1** | 2% | 35 : 35 : 10 : 10 | Fragmented | 12.8±2.08 | Excluded |
| **F2** | 5% | 35 : 35 : 10 : 10 | Intact | 19.1±2.03 | Selected |
| **F3** | 10% | 35 : 35 : 10 : 10 | Intact | 17.7±4.19 | Excluded |

***Note:***

^a^: OA content is calculated as a weight percentage of the final formulation (*W_0A_*/(*W_basal_*+*W_OA_*)×100%)

^b^: Represents the mass ratio of the basal matrix components (SPC : SMO : Ethanol : DMSO)

^c^: Assessed by visual observation after incubation in PBS (pH 7.4) at 37°C for 8 h.

^d^: Data represents mean ± SD (*n*=3).

**Figure S1**.Rheological amplitude sweep curves of the structurally dynamic LLC precursors. **(A)** Representative amplitude sweep profiles (storage modulus *G',* solid squares; loss modulus *G''*, open squares) for formulations F0–F3 at pH 7.4, demonstrating the progressive stiffening effect of OA. **(B)** Representative amplitude sweep profiles evaluating the material-payload synergy and pH-responsiveness: blank F2 matrix (pH 7.4), VDAP-loaded F2 matrix (pH 7.4), and VDAP-loaded F2 matrix (pH 5.0). All measurements were performed at a fixed frequency of 1 Hz at 37 °C. The data shown are representative curves from *n*=3 independent experiments, and the corresponding statistically extracted plateau *G'* values are summarized in Figure 2B and 2D of the main text.

**Figure S2. Steady-state shear rheological profiling of the optimized VDAP-LLC precursor.** (A) Flow curve showing shear stress as a function of shear rate. (B) Apparent viscosity versus shear rate profile. The continuous decrease in viscosity with increasing shear rate confirms the characteristic shear-thinning (non-Newtonian) property of the liquid precursor, providing the rheological basis for its smooth extrudability through fine needles. Data were acquired at 25 °C.

**Figure S3. Time-resolved polarized light microscopy (PLM) imaging of VDAP-LLC following medium exchange from pH 7.4 to pH 5.0.** VDAP-LLC initially displayed strong birefringent textures at 0 min. After exposure to pH 5.0, the birefringent domains progressively fragmented and weakened at 30 min, 1 h, and 6 h, and the field became predominantly optically isotropic at 24 h, with only weak residual birefringent textures. These optical changes are consistent with acid-triggered relaxation of the anisotropic *H*_II_-type organization toward a cubic-like mesophase. Scale bars: 100 μm.

**Figure S4. SAXS profiles of semaglutide-loaded LLC at pH 7.4, 6.5, and 5.0.** Semaglutide-loaded LLC shows ordered mesophase-associated diffraction peaks across the tested pH range; however, the normalized peak spacing ratios (relative to the lowest-*q* reflection) do not match the characteristic reflection sequence expected for a single canonical mesophase. Importantly, semaglutide-loaded LLC does not reproduce the pronounced pH-dependent topology remodeling observed for VDAP-LLC.

**Figure S5. Cumulative release profiles of semaglutide from semaglutide-loaded LLC at pH 7.4, 6.5, and 5.0 over 7 days.** Semaglutide exhibited relatively high cumulative released fractions across the tested pH range, with only modest enhancement upon acidification, and did not reproduce the pronounced pH-dependent retention-to-release transition observed for VDAP-LLC. Data are presented as mean ± SD (*n*=3)

**Figure S6. Gating strategy and flow cytometric analysis of apoptosis.** **(A-C)** Single-staining controls utilized for compensation and quadrant establishment: (A) Unstained cells (Blank), (B) Annexin V-FITC only, and (C) PI only. **(D-H)** Representative flow cytometry plots of 4T1 cells treated with VDAP (5, 25, and 100 μM) or DOX (1 μM) for 48 h. These plots correspond to the quantitative data presented in Figure 1C. Quadrants represent: necrotic cells (Q1, Annexin V^-^/PI^+^), late apoptotic cells (Q2, Annexin V^+^/PI^+^), early apoptotic cells (Q3, Annexin V^+^/PI^-^), and viable cells (Q4, Annexin V^-^/PI^-^).

**Figure S7. Flow cytometric gating strategy and representative histograms of surface Calreticulin (CRT).** **(A)**Hierarchical gating strategy employed for analysis. The population was first gated on FSC-H vs. SSC-H to exclude debris (left), followed by doublet discrimination using FSC-A vs. FSC-H (right) to isolate the single-cell population. **(B)** Representative histograms of surface CRT fluorescence intensity. 4T1 cells were treated with VDAP or Doxorubicin (DOX) for 16 h. The rightward shift in fluorescence intensity corresponds to the increased surface exposure of CRT relative to the untreated Control group.

**Figure S8. Quantification of ICD-associated DAMP biomarkers in tumor tissues.** Representative immunohistochemical staining and quantitative analysis of **(A)** HMGB1 and **(B)** HSP70 in tumor tissues. Data are presented as mean ± SD (*n* = 3). Statistical significance was determined using the Kruskal–Wallis test followed by Dunn’s multiple comparisons test. ^*P* < 0.05 and ^^*P* < 0.01vs. the VDAP-LLC group. Scale bars: 50 μm

**Figure S9. Gating strategies for flow cytometric analysis of immune populations.**

**(A)** Gating hierarchy for identifying splenic mature dendritic cells (DCs). Following doublet and dead cell exclusion, DCs were identified as CD45^+^ CD11c^+^ MHCII^+^. Mature DCs were further defined as CD80^+^ CD86^+^. **(B)** Gating hierarchy for identifying intratumoral cytotoxic T cells. Live, singlet CD45^+^ cells were gated for CD3^+^, followed by CD8^+^ selection. Activated cytotoxic T cells were defined as CD8^+^ IFN-γ^+^.
